# Genetic Architecture of Childhood Kidney and Urological Diseases in China

**DOI:** 10.1007/s43657-021-00014-1

**Published:** 2021-07-15

**Authors:** Ye Fang, Hua Shi, Tianchao Xiang, Jiaojiao Liu, Jialu Liu, Xiaoshan Tang, Xiaoyan Fang, Jing Chen, Yihui Zhai, Qian Shen, Guomin Li, Li Sun, Yunli Bi, Xiang Wang, Yanyan Qian, Bingbing Wu, Huijun Wang, Wenhao Zhou, Duan Ma, Jianhua Mao, Xiaoyun Jiang, Shuzhen Sun, Ying Shen, Xiaorong Liu, Aihua Zhang, Xiaowen Wang, Wenyan Huang, Qiu Li, Mo Wang, Xiaojie Gao, Yubin Wu, Fang Deng, Ruifeng Zhang, Cuihua Liu, Li Yu, Jieqiu Zhuang, Qing Sun, Xiqiang Dang, Haitao Bai, Ying Zhu, Siguang Lu, Bili Zhang, Xiaoshan Shao, Xuemei Liu, Mei Han, Lijun Zhao, Yuling Liu, Jian Gao, Ying Bao, Dongfeng Zhang, Qingshan Ma, Liping Zhao, Zhengkun Xia, Biao Lu, Yulong Wang, Mengzhun Zhao, Jianjiang Zhang, Shan Jian, Guohua He, Huifeng Zhang, Bo Zhao, Xiaohua LI, Feiyan Wang, Yufeng Li, Hongtao Zhu, Xinhui Luo, Jinghai Li, Jia Rao, Hong Xu

**Affiliations:** 1 Department of Nephrology, Children’s Hospital of Fudan University, National Pediatric Medical Center of CHINA, 399 Wanyuan Road, Shanghai, China; 2 Shanghai Kidney Development and Pediatric Kidney Disease Research Center, Shanghai, China; 3 Shanghai Key Lab of Birth Defect, Children’s Hospital of Fudan University, Shanghai, 201102 China; 4 Department of Rheumatology, Children’s Hospital of Fudan University, Shanghai, China; 5 Department of Urology, Children’s Hospital of Fudan University, Shanghai, China; 6 Clinical Genetic Center, Children’s Hospital of Fudan University, Shanghai, China; 7 Key Laboratory of Metabolism and Molecular Medicine, Ministry of Education, Department of Biochemistry and Molecular Biology, Institutes of Biomedical Sciences, School of Basic Medical Sciences, Fudan University, Shanghai, China; 8 The Children Hospital of Zhejiang University School of Medicine, Hangzhou, China; 9 The First Affiliated Hospital of Sun Yat-Sen University, Guangzhou, China; 10 Shandong Provincial Hospital, Jinan, China; 11 Bejing Children’s Hospital Affiliated to Capital University of Medical Science, Beijing, China; 12 Children’s Hospital of Nanjing Medical University, Nanjing, China; 13 Wuhan Children’s Hospital, Tongji Medical College, Huazhong University of Science and Technology, Wuhan, China; 14 Shanghai Children’s Medical Centre, Shanghai Jiaotong University School of Medicine, Shanghai, China; 15 Children’s Hospital of Chongqing Medical University, Chongqing, China; 16 Shenzhen Children’s Hospital, Shenzheng, China; 17 Shengjing Hospital of China Medical University, Shenyang, Liaoning China; 18 Anhui Provincial Children’s Hospital, Hefei, China; 19 Xuzhou Children’s Hospital, Xuzhou, China; 20 Henan Children’s Hospital, Zhengzhou, China; 21 Guangzhou First People’s Hospital, Guangzhou, China; 22 The Second Affiliated Hospital and Yuying Children’s Hospital of Wenzhou Medical University, Wenzhou, China; 23 Qingdao Women and Children’s Hospital, Qingdao, China; 24 Xiangya Hospital Central South University, Changsha, Hunan China; 25 The First Affiliated Hospital of Xiamen University, Xiamen, China; 26 First Affiliated Hospital of Anhui Medical University, Hefei, China; 27 Children’s Hospital of Lianyungang, Lianyungang, China; 28 Tianjin Children Hospital, Tianjing, China; 29 The Children’s Hospital of Guiyang City, Guiyang, China; 30 Qilu Children’s Hospital of Shandong University, Jinan, China; 31 Dalian Children’s Hospital, Dalian, China; 32 Shanxi Children’s Hospital, Taiyuan, China; 33 Boai Hospital of Zhongshan, Zhongshan, China; 34 Weifang Maternal and Child Health Hospital, Weifang, China; 35 Xi’an Children’s Hospital, Xian, China; 36 Children’s Hospital of Hebei Province, Shijiazhuang, China; 37 First Affiliated Hospital of Jilin University, Changchun, China; 38 Wuxi Municipal Children’s Hospital, Wuxi, China; 39 Department of Pediatrics, Jinling Hospital, Nanjing Medical University, Nanjing, China; 40 General Hospital of Ningxia Medical University, Yinchuan, China; 41 The Second Hospital of Shandong University, Jinan, China; 42 Shenzhen Hospital of University of Hong Kong, Shenzhen, China; 43 First Affiliated Hospital of Zhengzhou University, Zhengzhou, China; 44 Peking Union Medical College Hospital, Beijing, China; 45 Child Health Hospital of Foshan, Foshan, Guangdong China; 46 The Second Hospital of Hebei Medical University, Shijiazhuang, China; 47 Kunming Children’s Hospital, Kunming, China; 48 Affiliated Hospital of Inner Mongolia Medical University, Hohehot, China; 49 Urumqi City Children’s Hospital, Urumqi, China; 50 Xinhua Hospital Affiliated to Medical College of Shanghai Jiaotong University, Shanghai, China; 51 Academy of Pediatrics, Xinjiang Medical University, Urumqi, China; 52 Xinjiang Uygur Autonomous Region People’s Hospital, Urumqi, China; 53 Changchun Children’s Hospital, Changchun, China

**Keywords:** Chronic kidney disease (CKD), Exome sequencing (ES), Steroid-resistant nephrotic syndrome (SRNS), Congenital anomalies of the kidney and urinary tract (CAKUT), Nephronophthisis (NPHP), Polycystic kidney disease (PKD)

## Abstract

Kidney disease is manifested in a wide variety of phenotypes, many of which have an important hereditary component. To delineate the genotypic and phenotypic spectrum of pediatric nephropathy, a multicenter registration system is being implemented based on the Chinese Children Genetic Kidney Disease Database (CCGKDD). In this study, all the patients with kidney and urological diseases were recruited from 2014 to 2020. Genetic analysis was conducted using exome sequencing for families with multiple affected individuals with nephropathy or clinical suspicion of a genetic kidney disease owing to early-onset or extrarenal features. The genetic diagnosis was confirmed in 883 of 2256 (39.1%) patients from 23 provinces in China. Phenotypic profiles showed that the primary diagnosis included steroid-resistant nephrotic syndrome (SRNS, 23.5%), glomerulonephritis (GN, 32.2%), congenital anomalies of the kidney and urinary tract (CAKUT, 21.2%), cystic renal disease (3.9%), renal calcinosis/stone (3.6%), tubulopathy (9.7%), and chronic kidney disease of unknown etiology (CKDu, 5.8%). The pathogenic variants of 105 monogenetic disorders were identified. Ten distinct genomic disorders were identified as pathogenic copy number variants (CNVs) in 11 patients. The diagnostic yield differed by subgroups, and was highest in those with cystic renal disease (66.3%), followed by tubulopathy (58.4%), GN (57.7%), CKDu (43.5%), SRNS (29.2%), renal calcinosis /stone (29.3%) and CAKUT (8.6%). Reverse phenotyping permitted correct identification in 40 cases with clinical reassessment and unexpected genetic conditions. We present the results of the largest cohort of children with kidney disease in China where diagnostic exome sequencing was performed. Our data demonstrate the utility of family-based exome sequencing, and indicate that the combined analysis of genotype and phenotype based on the national patient registry is pivotal to the genetic diagnosis of kidney disease.

**Supplementary Information:**

The online version contains supplementary material available at 10.1007/s43657-021-00014-1.

## Background

Chronic kidney disease (CKD) is a broad term that encompasses many complex disorders that collectively affect over 1 in 10 individuals worldwide, resulting in substantial morbidity and mortality as well as the high healthcare costs. Recent predictions from the Institute of Health Metrics and Evaluations indicated that CKD will be the fifth leading cause of global mortality by 2040 (Jager et al. 2019; Foreman et al. 2018). In addition to Mendelian nephropathy, multiple lines of evidence support a wider genetic contribution to the pathogenesis of CKD. 10–29% of adults with chronic kidney failure have a positive family history of kidney disease across different ethnic groups and etiologies. Furthermore, glomerular filtration rate has a heritability of approximately 30–60% in the general population (Saran et al. 2020; Connaughton et al. 2019), and other indices of kidney function, such as albuminuria and electrolyte excretion, show similar significant heritability (Stevens et al. 2013; Hays et al. 2020). It is important for all those involved in either the care of patients with kidney disease or the study of the kidney to have a basic understanding of the spectrum of variation and the breadth of phenotypes that are relevant to kidney health and disease.

Pediatric patients with kidney disease, particularly the young patients, may have nonspecific signs and symptoms that are unrelated to the urinary tract. Early diagnosis and treatment of childhood kidney disease play an important role in the prevention of CKD. In recent years, next-generation sequencing has provided us with a better understanding of the genetic landscape of CKD in children and young adults. Approximately 500 monogenic causes of CKD have been identified (Hildebrandt 2016). However, the phenotypes associated with genetic forms of kidney disease can be highly variable and may overlap, which can complicate diagnosis based solely on clinical symptomatology. Phenotypic heterogeneity in the clinical expression of nephropathy poses challenges for the follow-up, management, and treatment of pediatric patients (Groopman et al. 2020). Limited sources of phenotypic complexity are found in rare genetic nephropathies in China. A national multicenter registration network (Chinese Children Genetic Kidney Disease Database, CCGKDD, www. ccgkdd.com.cn) was developed in 2014 and formally launched in 2017. Led by local experts from the National Pediatric Medical Center of China, the database integrates clinical information and genetic detection from 147 hospitals in the nation (see “Online Resource 1”). The CCGKDD cohort provides a unique opportunity to map the genetic spectrum of kidney diseases in China. Hence, we report the joint analysis of phenotype and genotype to unravel the genetic architecture and phenotypic heterogeneity of kidney and urological diseases.

## Methods

### Study Design and Participants

**Fig. 1:**
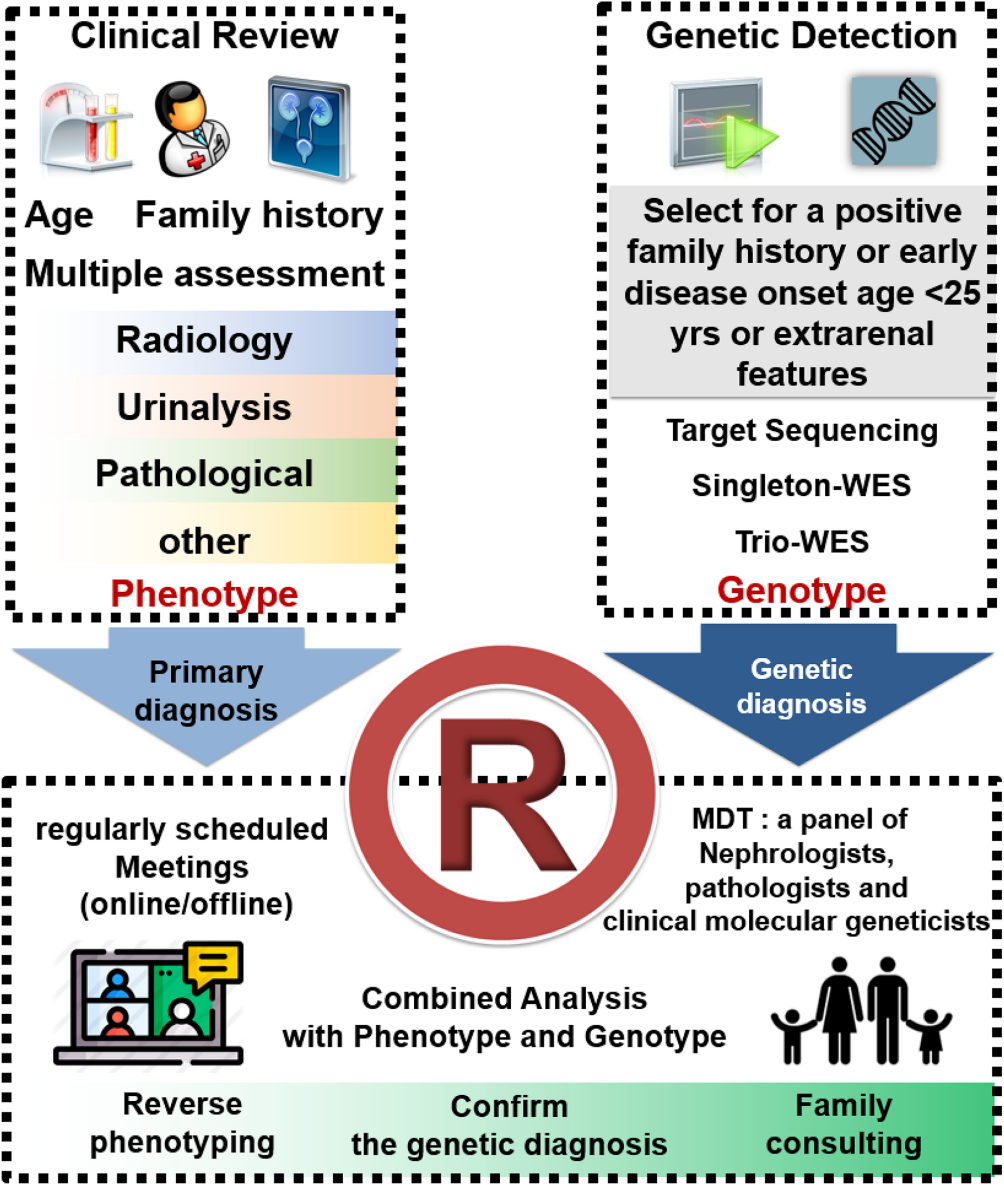
The workflow of this study. The multidisciplinary team (MDT) consists of nephrologists, pathologists, clinical geneticists, and genetic counsellors from different leading medical centers. Patients attend a local clinic with an appointment for clinical review. Patients who are less than 25 years old (yrs) with a positive family history or early-onset kidney disease or extrarenal features are encouraged to receive genetic detection, and the consent was previously obtained from each guardian of the patients. Blood samples were taken from the local hospitals for sequencing, and the clinically accredited genomic sequencing was performed. Bioinformatic analysis was conducted initially by local clinical geneticists, then the information on phenotype and genotype is uploaded to the registry for re-evaluation by the team from Children’s Hospital of Fudan university (central unit). The data were reviewed to reach the consensus to ensure that the genotype is consistent with the phenotype. The reports of genetic diagnosis and reverse phenotyping are returned to the patients and their families. See “Methods” and “Online Resource 2” for details

Children and adolescents with kidney and urological diseases from 0 to 18 years of age were prospectively recruited between January 2014 and November 2020 based on the data from the CCGKDD. The Institutional Review Board (IRB) of the Children’s Hospital of Fudan University approved and supervised this study involving the participating centers (NO. 2018_286). The eligibility requirements for registration included comprehensive phenotype and genotype information of each family. Each health center identified the eligible patients after the standardized training from “Internet Plus” Nephrology Alliance of National Center for Children’s Care. The features of patients includ: (1) a family history of kidney disease, defined as any lineal relatives by blood or collateral relatives by blood up to three generations who were diagnosed with urinary abnormalities or impaired kidney function, as reported by their guardians; (2) functional and structural abnormalities of the kidney and urinary tract screening out the secondary findings (e.g. long-term history of diabetes mellitus before ESRD, systemic lupus erythematosus, IgA nephropathy or Schonlein-Henoch purpura nephropathy verified using biopsy, acquired obstructive uropathy, tumor, etc.); (3) clinical suspicion of genetic kidney disease due to childhood early-onset CKD; (4) extrarenal features. The recruitment process is shown in Fig. 1. These families were referred for assessment and management of kidney diseases and consented to general genetic research.

### Phenotyping

Upon registration, genetic counselors generated a three-generation pedigree based on the family history reported by the guardians of the proband, and the data were collected from all centers in a unified and anonymous database. Clinical experts in pediatric nephrology identified the phenotype data. The phenotypic information for registration was collected and checked by the trained data entry clerks who only had the registration ID number to confirm the individual information prior to the final release of the registry. Parental health records were not available for the study and a physical examination was not conducted. The questionnaire on family history was completed following the clinical interview of the physicians with the guardians. The authors attested to the accuracy and comprehensiveness of the data present in the database (Online Resource 2).

The initial clinical features were divided into four subgroups: abnormal urinalysis results, abnormal radiological examination results, abonrmal renal biopsy results, and other phenotypes with normal urinalysis/radiology/histology results. The primary clinical diagnosis of each patient was determined by a medical history review and the referral of primary nephrologists in one of the following *priori* clinical diagnostic categories (Connaughton et al. 2019; Venkat-Raman et al. 2012):

- Glomerulonephritis (GN): presenting with hematuria and/or non-nephrotic proteinuria, encompassing membranoproliferative GN, crescentic GN, and hemolytic uremic syndrome (HUS);
- Steroid-resistant nephrotic syndrome (SRNS), or nephrotic syndrome with biopsy findings of FSGS;
- Congenital anomalies of the kidney and urinary tract (CAKUT) are defined as any abnormality of number, size, shape, or anatomic position within the kidneys or urinary tract;
- Cystic kidney disease including nephronophthisis (NPHP), medullary cystic disease, and other renal cystic ciliopathies;
- Renal tubular disease, including clinical diagnosis of tubulopathy and tubulointerstitial nephritis confirmed by renal biopsy (TK);
- Nephrolithiasis and renal calcinosis;
- CKD of unknown etiology (CKDu): based on the definition of CKD of unknown etiology (Wijewickrama et al. 2019).

### Exome Sequencing and Variant Information

Samples of affected individuals were subjected to the exome sequencing (ES) of parent–child trios upon obtaining the informed consent. The annotation of the ES procedure and its variants has been described in detail previously (Online Resource 2) (Groopman et al. 2019). Variant interpretation was performed manually by a panel of nephrologists and clinical molecular geneticists. For clinical sequence interpretation, variants were classified according to the American College of Medical Genetics and Genomics (ACMG) guidelines (Richards et al. 2015). All the information on the pathogenic variants and variants of uncertain significance (VUS) of known pathogenic genes can be found on the website of www. ccgkdd.com.cn with a guest account (browse only, not for download).

### Reverse Phenotyping

We systematically defined the disease-causing genes and the phenocopy genes. A disease-causing gene was a gene known to be associated with a clinical phenotype based on the Human Gene Mutation Database (HGMD), OMIM-Morbid database or the medical literature. A phenocopy is defined as a situation in which a patient has a phenotype that corresponds to a specific hereditary disease without detection of the expected genotype but with detection of a different genotype (Mariani et al. 2016). To perform reverse phenotyping, we established a multidisciplinary team composed of at least one geneticist, one nephrologist and one nephropathologist, discussing clinical cases of patients undergoing ES during regularly scheduled meetings (Fig. 1). Patients with pathogenic variants of phenocopy genes and their families were also thoroughly examined with the help of external consultants, based on the clinical indication obtained upon genetic test. In a general way, geneticists illustrated the suspicious variants and correlated their data with existing literatures. Nephrologists proposed a list of clinical examinations and expert consultations to detect neglected signs or symptoms of syndromic genetic disorders suggested by genetic analysis. In addition, pathologists proposed whether and how to re-evaluate the kidney biopsy, looking for specific signs of suspected genetic nephropathy, including subsequent staining.

## Results

**Fig. 2:**
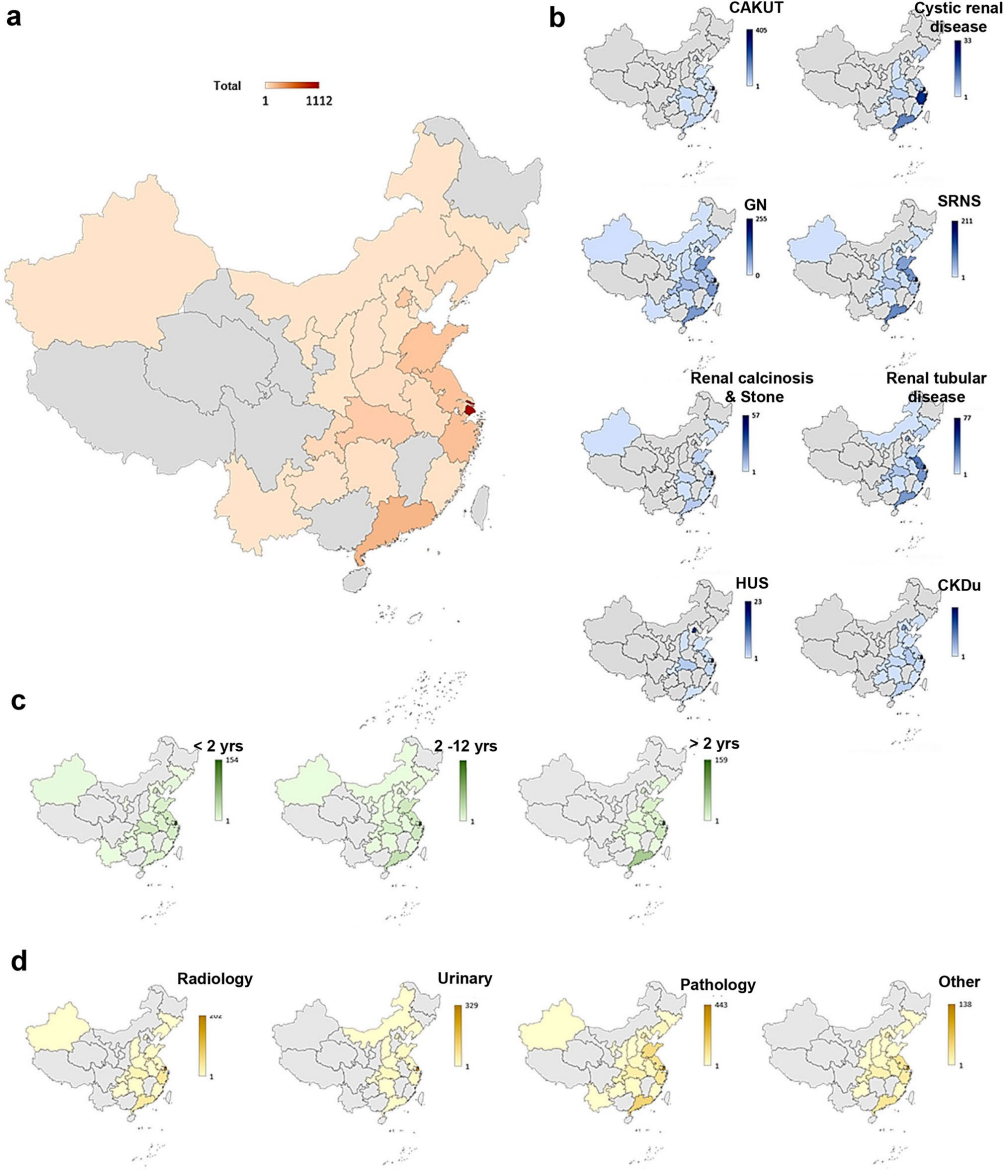
The geographical distribution of pediatric patients with kidney disease enrolled in the Chinese Children Genetic Kidney Disease Database (CCGKDD). Provinces are colored according to the number proportion of the patients. **a** The geographical distribution of total patients from different provinces. **b** The geographical distribution of eight subgroups with different clinical primary diagnosis, including congenital anomalies of the kidney and urinary tract (CAKUT), cystic kidney disease, Glomerulonephritis (GN), steroid-resistant nephrotic syndrome (SRNS), renal calcinosis and stone, renal tubulopathy, hemolytic uremic syndrome (HUS) and CKD of unknown etiology (CKDu). **c** The geographical distribution of three different age groups including the group of less than 2 years old, the group between 2 and 12 years old, and the group of more than 12 years old. **d** The geographical distribution of four subgroups with different initial phenotypes, including abnormal radiological images (Radiology), abnormal urinalysis (Urinalysis), abnormal pathological findings (Pathology) and other phenotypes (Other). The differences between colors shown in each panel are equal. yrs, years old

A total of 2256 affected individuals (the ratio of the number of males to the number of females was 1.6:1) from 2254 families were recruited with records in CCGKDD between 2014 and 2020. Consanguinity was observed in eight families. All the information of the 2256 patients came from 51 medical centers of 23 provinces in China. The demographic data and the geographic distribution of cases are shown in Fig. 2. There were 77 cases registered in 2014, 192 cases in 2015, 281 cases in 2016, 254 cases in 2017, 198 cases in 2018, 691 cases in 2019 and 563 cases in 2020 (Online Resource 3).

### Clinical Characteristics

**Fig. 3:**
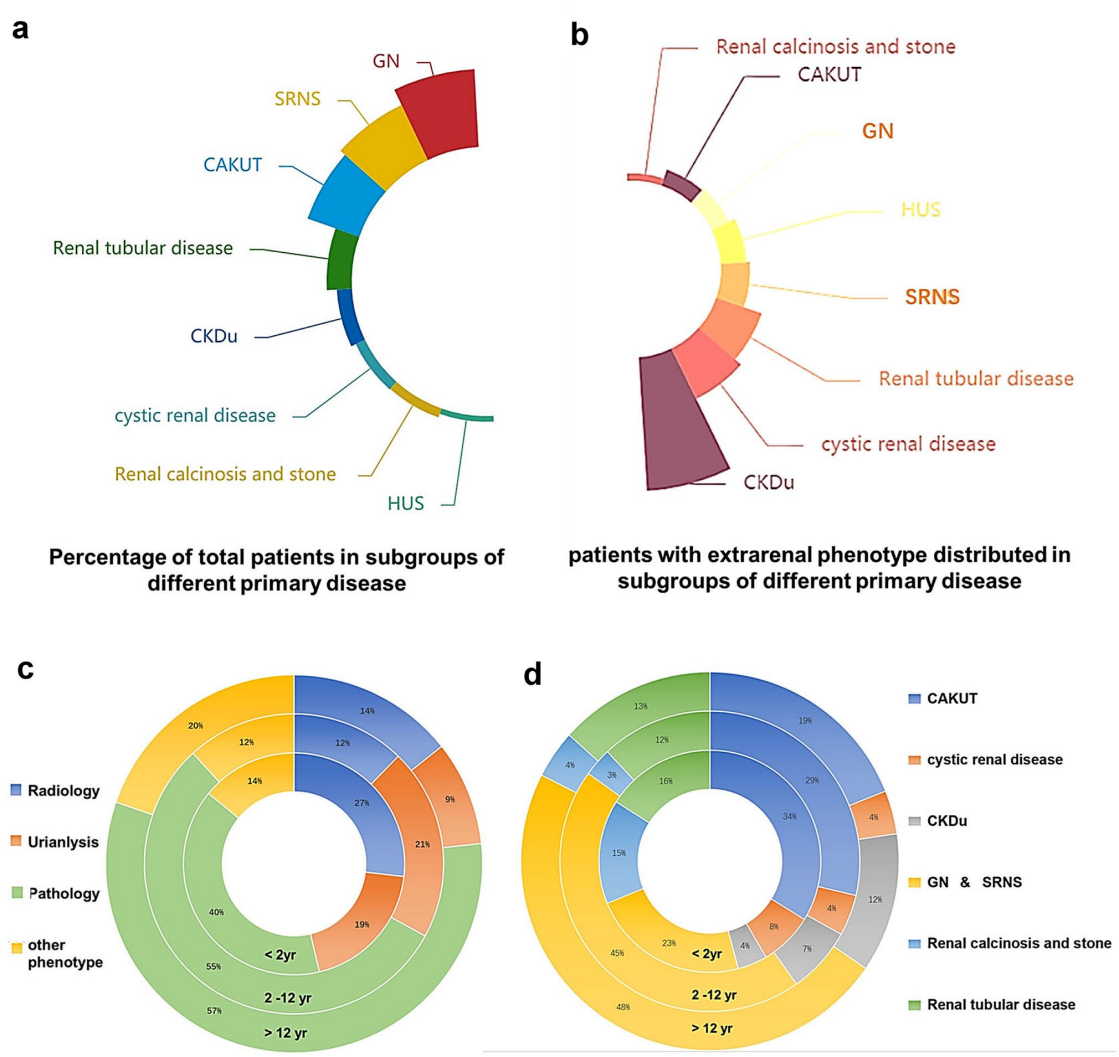
Distribution of patients in each subgroup with different phenotypes. **a** Percentage distribution of the total patients in subgroups of different primary diagnosis. **b** Percentage distribution of the patients with extrarenal phenotype in subgroups of different primary diagnosis. **c** Percentage distribution of the patients of different age that the genetic sequencing was performed in subgroups of different initial phenotypes, including abnormal radiological images (Radiology), abnormal urinalysis (Urinalysis), abnormal pathological findings (Pathology), and other phenotypes (Other). **d** Percentage distribution of the patients of different age that the genetic sequencing was performed in subgroups of different primary diagnosis

Phenotypic profiling showed that the primary clinical diagnoses included SRNS (23.5%, 531/2256), GN (32.2%, 726/2256), CAKUT (21.2%, 478/2256), cystic renal disease (3.9%, 89/2256), renal calcinosis or stone (3.6%, 82/2256), renal tubular disease (9.7%, 219/2256), and CKDu (5.8%, 131/2256) (Fig. 3a). A total of 53.0% (1195/2256) of the probands were initially diagnosed based on the pathological findings. Moreover, 27.7% (625/2256) of the patients had the abnormalities detected using ultrasound or other radiological examinations, including voiding cystourethrography (VCUG) as the gold standard diagnostic test for vesicoureteral reflux (VUR). Cases of VUR were reported in 13.3% (301/2256) of the pediatric patient cohort. Abnormal urinalysis results, including hematuria, proteinuria or pyuria were reported in 71.8% (1618/2224) of patients. Among the patients with CKDu , 80.9% (106/131) of them were initially diagnosed based on renal function deficiency without any abnormal findings on radiological examination or urinalysis.

Extrarenal phenotypes were observed in 138 patients in our study. Patients with an extrarenal phenotype were distributed with a wide range of kidney diseases. Note that 22.9% of patients with CKDu had the extrarenal phenotypes, which showed significantly greater predominance than that seen in the other patient groups (Fig. 3b). Among the patients with extrarenal symptoms, 22.4% had neurological disorders, 13.0% had cardiological disorders, 69.4% had vision problems, 5.1% had hearing loss, and 4.3% had skeletal deformities.

According to the age distribution of the patients who participated in genetic sequencing performed in this study, 14.2% of patients were younger than 2 years old, 70.5% of patients were between 2 and 12 years old, and 15.2% of patients were more than 12 years old. More cases of CAKUT or cystic kidney disease were reported in the younger age group who participated earlier in the genetic sequencing. The patients less than 2 years of age who participated in genetic sequencing were oberversed with higher percentage with abnormal radiological findings than that with abnormal findings on urinalysis or renal histology (*Fisher’s exact test,* *P* < 0.05, Fig. 3c, d).

Geographical distribution of patients showed that pediatric patients who participated in genetic studies for kidney disease in our registry were mainly distributed in Eastern China. The regions with the highest proportion of patients with CAKUT or cystic kidney disease were Eastern China and Central China, respectively. However, few records from Western China were available in genetic findings of the patients with CAKUT, cystic renal disease or HUS (Fig. 2).

### Genetic Findings and Diagnostic Yield

**Fig. 4:**
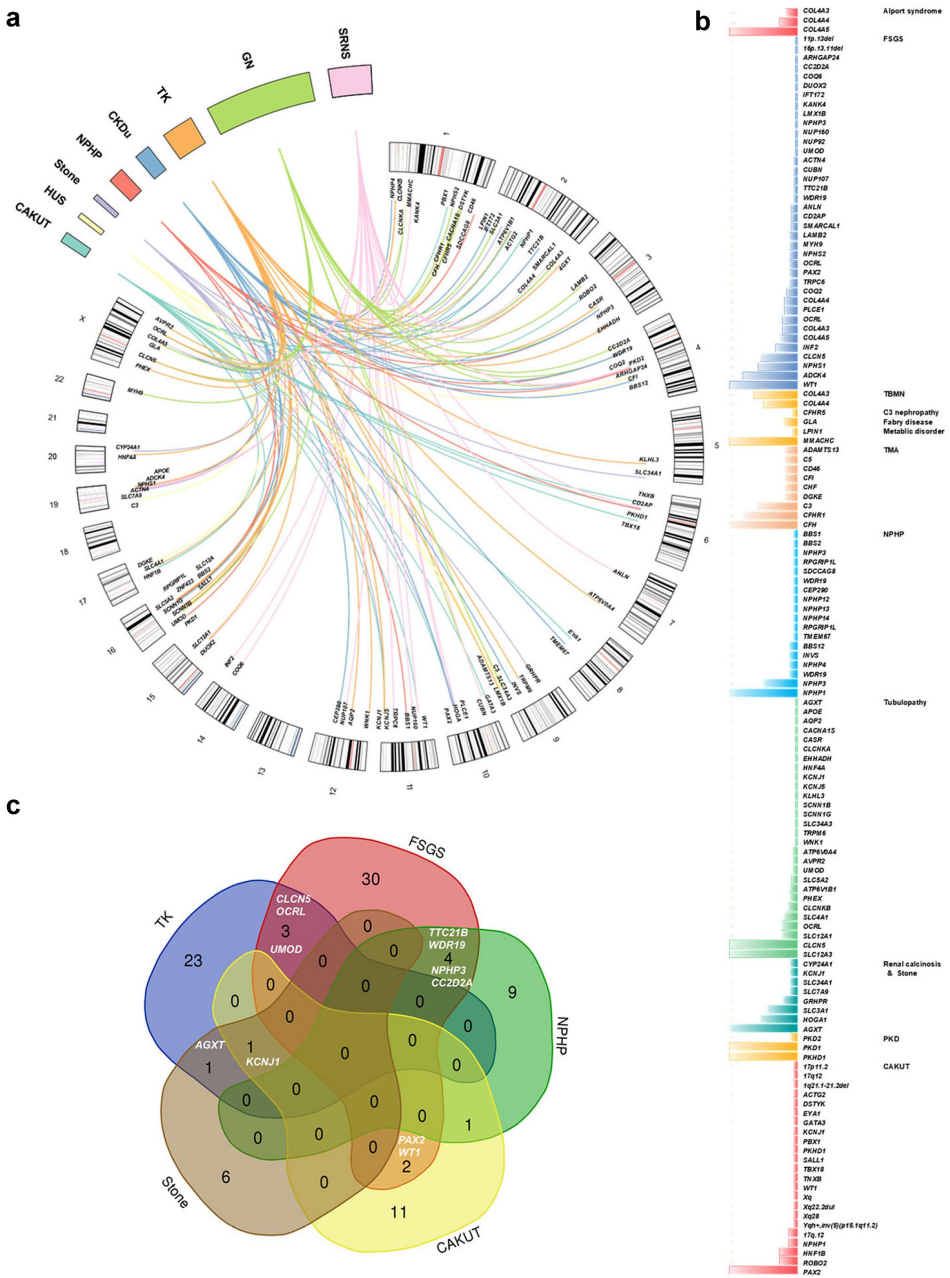
Genetic spectrum in 883 pediatric patients with kidney and urological disease out of the 2256 children enrolled in the registry of CCGKDD from 2014 to 2020. **a** Circos-style plot of genetic diagnosis in 883 patients with kidney disease. Eight categories of primary diagnosis of kidney diseases are indicated outside the widest arc of the circle, chromosome numbers are labeled outside the smaller arc, and gene symbols (patients with pathogenic or likely pathogenic variants) are listed inside. Links are colored into eight categories. **b** Genetic diagnoses post exome sequencing are shown with colored columns indicating the case number of patients for identifying the pathogenic variants in different genes, and different subgroups of diagnosis are labelled with different color in columns. **c** Venn diagrams displaying the overlap among the causative genes of kidney diseases

Molecular genetic diagnoses were performed in 39.1% (883/2256) of the pediatric cohort with kidney disease. Of the 105 monogenetic disorders identified in this study (Fig. 4, Online Resource 4), there were pathogenic or likely pathogenic variants of 46 genes accounted for 228 genetic diagnoses of autosomal dominant (AD) diseases, 53 genes accounted for 293 genetic diagnoses of autosomal recessive (AR) diseases, and six genes accounted for 351 genetic diagnoses of X-linked (XL) diseases. Additionally, 10 distinct genomic disorders were detected through ES, and subsequently confirmed as pathogenic copy number variants (CNVs) in 11 patients. No patient received a dual or multiple molecular diagnosis, in which multiple significant findings were associated with non-overlapping clinical presentations or could possibly contribute to the major phenotypes.

**Fig. 5:**
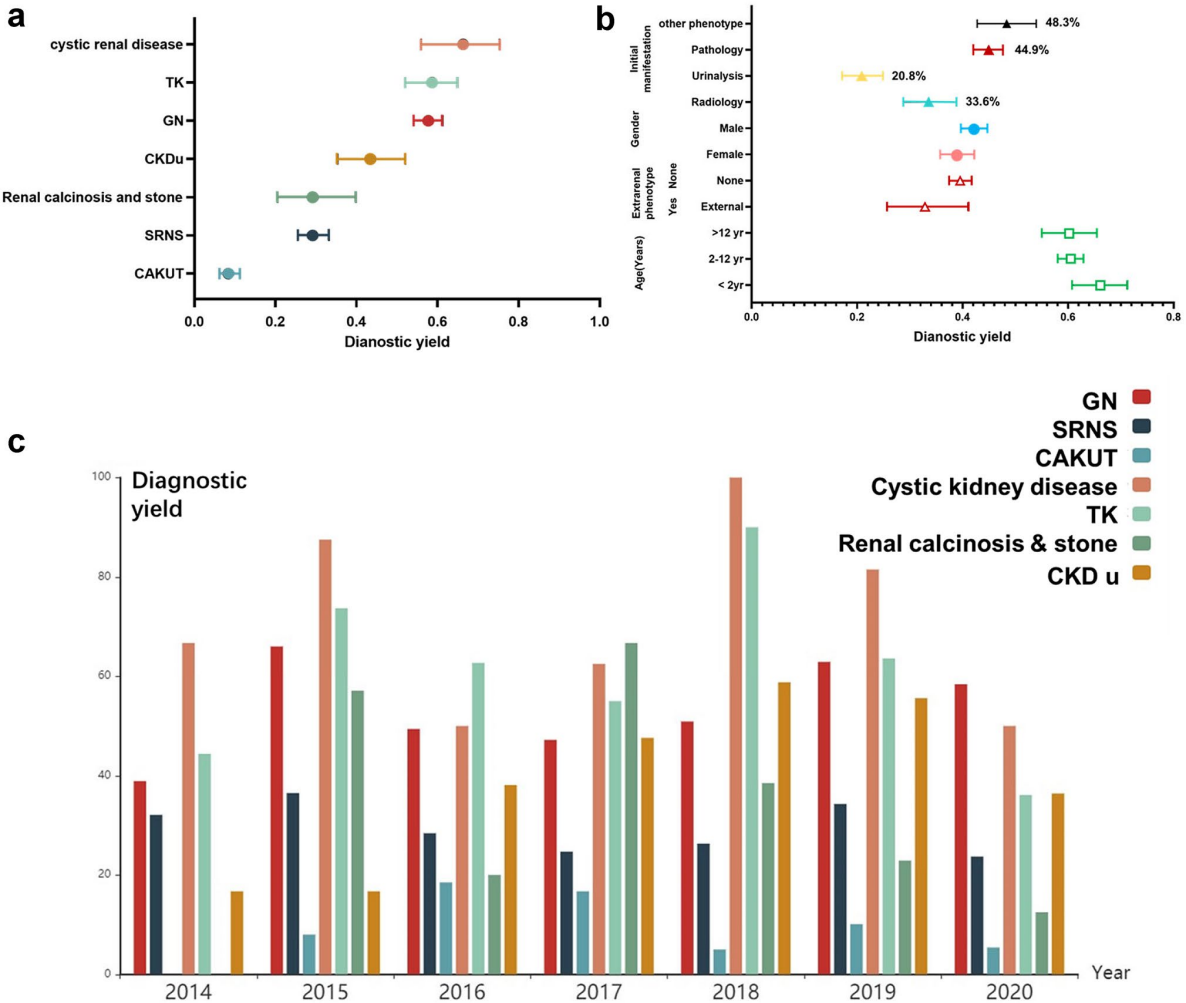
Genetic test yields in children with different category of renal disease. **a** Diagnostic ratio in patient groups of different primary diagnosis. **b** Diagnostic ratio in subgroups of different initial phenotypes, including abnormal radiological images (Radiology), abnormal urinalysis (Urinalysis), abnormal pathological findings (Pathology), and other phenotypes (Other), and diagnostic ratio in subgroups of different sex, presence of extrarenal phenotype, or age at initial presentation. **c** Diagnostic ratio in patient groups with different registration year. Vertical bars indicate 95% CI

The diagnostic yield differed by diagnostic subgroups (*P* < 0.001), and was highest in those with cystic renal disease (66.3%), followed by tubulopathy (58.4%), GN (57.7%), CKDu (43.5%), SRNS (29.2%), renal calcinosis and stone (29.3%) and CAKUT (8.6%). The diagnostic ratio of the patient group with abnormal pathological findings was higher than that of the patient group with abnormal urinalysis or abnormal radiological results (44.9% vs. 20.8%, 44.9% vs. 33.6%, *P* < 0.001). In the patient group with no abnormal findings upon urinalysis, radiological or pathological detection, the ratio of genetic diagnosis was 42.7%, which was not significantly different from that of patients with abnormal pathological findings (*P* > 0.05). Among patients with extrarenal features, monogenic causality was observed in 33.3% (46/138) of the patients. No significant differences were observed in the diagnostic ratio of the patient group with extrarenal features compared to the patient group without extrarenal features (33.3% vs. 39.55%, *P* > 0.05). There was no association between the sex, presence of extrarenal manifestations, or age at the initial presentation with a positive diagnosis (Fig. 5b). No significant differences in the genetic diagnostic ratio were observed among the different patient groups at various registration time (Fig. 5c).

### Genetic Analysis Modified the Diagnosis of Kidney and Urological Disease

ES confirmed a specific underlying cause within the broader category of a clinically suspected disease in 786 patients, corrected the a *priori* clinical diagnosis in 40 patients, and established a diagnosis for 57 patients with CKDu (Fig. 4, Online Resource 4).

In 677 patients with a *priori* clinical diagnosis of GN, pathogenic variants in *COL4A5*, *COL4A3* or *COL4A4* were detected confirming the diagnosis of Alport syndrome (345) or TBMN (13). ES also provided the genetic findings that modified the diagnosis in 29 patients with GN. The molecular genetic diagnoses in these patients included the following: Dent disease (*CLCN5*, n = 11), Lowe syndrome (*OCRL*, n = 4), methylmalonic aciduria with homocysteinemia CblC type (*MMACHC*, n = 7), NPHP (*NPHP3*, n = 1; *WDR19*, n = 1; *CC2D2A*, n = 1; *IFT172*, n = 1), Fabry disease (*GLA*, n = 2), monogenic podocytopathies (*NPHS2*, n = 1; *MYH9*, n = 1; *COQ2*, n = 1; *LAMB2*, n = 1; *CUBN*, n = 1; *LMX1B*, n = 1; *UMOD*, n = 1; *TTC21B*, n = 1), C3 nephropathy (*CFHR5*, n = 1). Additionally, pathogenic variants were identified in 19 out of 49 cases referred to HUS.

For patients with SRNS, pathogenic variants in 30 monogenic causalities were confirmed in 155 of the 531. The most frequently pathogenic genes were *WT1* (31)*, COQ8B* (22)*, NPHS1* (18)*, INF2* (10) and *PLCE1* (6). We corrected the diagnosis of four cases of Alport syndrome (*COL4A5,* n = 1), Lowe syndrome (*OCRL*, n = 2) and Dent disease (*CLCN5*, n = 1). In addition, CNV in loci *11p.13* and *16p.13* were detected in two cases of SRNS accompanied by renal dysplasia, and mental retardation. Polydactyly was found in one of them with CNV at the *16p.13* locus. In families with an a *priori* clinical diagnosis of cystic nephropathy (89 of 2256), we detected a pathogenic variant in 59 of 89 (66.3%) patients. The molecular genetic diagnosis confirmed the pre-ES clinical diagnosis, with detection of pathogenic variants in cystic kidney disease or nephronophthisis genes, including *PKD1* (25)*, PKD2* (3)*, PKHD1* (25)*, NPHP3* (1)*, WDR19* (1)*, RPGRIP1L* (1)*, BBS1* (1)*, BBS2* (1), and *SDCCAG8* (1).

For patients with CAKUT (447 of 2256), pathogenic variants were detected in 41 of 478 (9.1%). Twenty-eight patients had pathogenic variants in monogenic disease-causing genes, including *PAX2* (12), *ROBO2* (4), *HNF1B* (3), *DSTYK* (1), *EYA1* (1), *GATA3* (1), *PBX1* (1), *SALL1* (1), *TBX18* (1), and *TNXB* (1). In four families clinically diagnosed with CAKUT, pathogenic variants were found in genes other than the established CAKUT genes (*WT1*, n = 1; *PKHD1,* n = 1; *NPHP1*, n = 2). Genomic disorders were confirmed in nine patients with CAKUT. The *17q12* locus was identified in three families with a kidney anomaly phenotype. The remaining five loci (*1q21, 5p15.1, 17p11.2, Xq22.2* and *Xq28*) were found in six other CAKUT cases with mental retardation and growth retardation.

For patients with tubulopathy, pathogenic variants were detected in 28 known pathogenic genes in 128 of the 219. The molecular genetic diagnoses in these patients included the following: Dent disease (*CLCN5,* n = 36), Lowe syndrome (*OCRL*, n = 7), Gentleman syndrome (*SLC12A3,* n = 33), renal tubular acidosis (*SLC4A1*, n = 7; *ATP6V1B1*, n = 4; *ATP6V0A4*, n = 2; *EHHADH*, n = 1; *WNK1*, n = 1), Batter syndrome (*SLC12A1*, n = 8; *CLCNKB*, n = 5; *CLCNKA*, n = 1; *SCNN1B*, n = 1; *CACNA1S1*, n = 1), Fanconi syndrome (*HNF4A*, n = 1; *SLC34A3*, n = 1; *SLC4A1*, n = 1; *APE*, n = 1), hypophosphatemic rickets (*PHEX*, n = 4), nephrogenic diabetes insipidus (*AQP2*, n = 1; *AVPR2*, n = 2), renal diabetes (*SLC5A2*, n = 3), Liddle syndrome (*SCNN1G*, n = 1), primary hypomagnesemia (*TRPM6*, n = 1), ADTKD (*UMOD*, n = 1), renal calcinosis (*KCNJ1*, n = 1; *KCNJ5*, n = 1) and hyperoxaluria (*KLHL3*, n = 1;  *AGXT*, n = 1). In 24 of the 82 patients referred with nephrolithiasis and renal calcinosis, pathogenic variants in eight disease-causing genes were detected, including *AGXT* (9), *HOGA1* (5), *SLC3A1* (4), *KCNJ1* (1), *GRHPR* (2), *CYP24A1* (1), *SLC34A1* (1) and *SLC7A9* (1).

In patients with CKDu (131 of 2256, 5.8%), pathogenic variants were detected in 57 of 131 (43.5%). Molecular genetic diagnoses in these families included the following: NPHP (*NPHP1*, n = 16; *NPHP3*, n = 2; *INVS*, n = 2; *NPHP4*, n = 2; *WDR19*, n = 2;  *BBS12*, n = 2;  *NPHP13*, n = 1; *RPGRIP1L*, n = 2; *TMEM67*, n = 1), methylmalonic aciduria with homocysteinemia CblC type (*MMACHC*, n = 7), FSGS (*COQ8B*, n = 3; *COQ2*, n = 2), Alport syndrome (*COL4A5*, n = 1), ADTKD (*UMOD*, n = 1), Lowe syndrome (*OCRL*, n = 1), renal dysplasia (*PAX2*, n = 2; *INF1B*, n = 1) and rhabdomyolysis syndrome (*LPIN1*, n = 1).

### Reverse Phenotyping

Reverse phenocopying following ES identified pathogenic variants, adding a diagnosis ratio of 1.8% (40/2256) of patients with a clinical diagnosis of GN (29), SRNS (4), CAKUT (2), and TK (5). Clinical reassessment of these patients with unexpected genetic findings and their families led to the identification of minor or overlooked signs referring to the genetic diseases suggested by ES. These signs and symptoms were either present at the time of referral or appeared later during the follow-up processes. The diagnosis was corrected for these patients at a median age of 8.2 years. Reverse phenotyping allowed correct identification of 15 patients with Dent disease, six patients with Lowe syndrome, five patients with methylmalonic aciduria (CblC type), five patients with NPHP, two patients with Fabry disease, one patient with Alport syndrome, one patient with C3 nephropathy, one patient with hyperoxaluria, one patient with rhabdomyolysis syndrome, one patient with *UMOD*-associated FSGS, one patient with *TTC21B*-associated FSGS and one patient with *APE*-associated Lipid nephropathy, respectively.

## Discussion

We explored the genotype and phenotype spectrum of kidney and urological diseases in a national cohort of 2256 Chinese children. Patients were recruited from the CCGKDD, which is the largest national multicenter registration system for the genetic diagnosis of renal disease in China. In the CCGKDD cohort, the genetic diagnosis was confirmed in 39% of patients with 105 monogenetic diseases and 10 genomic diseases. An increasing number of medical centers and physicians from different regions of China have joined the national multicenter registration network, building local teams for genetic diagnosis of kidney disease. Therefore, there has been a marked increase in the number of reported cases from 2014 to 2020.

ES has improved the precision of diagnosis in patients with kidney diseases. Diagnosis yield is higher in patients with a positive family history of CKD, extrarenal manifestations, or a history of consanguinity (Groopman et al. 2019; Vivante and Hildebrandt 2016; Chirita-Emandi et al. 2020). A genetic study in a broad CKD population found that nephropathy of unknown origin, a family history of CKD, and clinical diagnosis of cystic renal disease were independent predictors of a genetic diagnosis (Groopman et al. 2019). Children with CKD are more likely to have an inherited form of kidney disease. Approximately 20% of early-onset CKD patients (i.e. before the age of 25) have a genetic basis for kidney disease (Vivante and Hildebrandt 2016). Genetic testing can identify the causes of disease regardless of clinical stage, contrary to diagnostic renal biopsies that often fail to diagnose the disease at very early or late stages (Groopman et al. 2020). In our cohort, molecular diagnosis was confirmed in 42.7% of the patients who did not show any abnormal findings on urinalysis, radiological or pathological detections. This was comparable to the diagnostic yield of patients with abnormal pathological findings upon renal biopsy. Furthermore, we detected pathogenic variants in 43.5% of patients with CKDu. The genetic diagnostic ratio among different age groups of patients was not significantly different. Genetic analysis can be a valuable tool for the diagnosis of patients with an unknown etiology or patients with an atypical or nonspecific clinical presentation. Patients with an underdiagnosed primary disease would benefit from an early  diagnosis, which could permit early intervention to reduce the risk of complications and slow down the progression of CKD (Hays et al. 2020; Groopman et al. 2020).

The most frequent causes of childhood CKD are CAKUT, glomerulonephritis, SRNS, and renal ciliopathies (NPHP). These kidney diseases may all have a genetic etiology. Our study examined the diagnostic yield of family-based ES in pediatric cohorts with a specific phenotype. We identified a genetic cause in 21% of patients with CAKUT. The most frequently mutated genes were *PAX2*, *ROBO2* and *HNF1B,* which accounted for 46% of the solved cases with CAKUT. Pathogenic CNVs were detected in 2% of CAKUT patients. A study on CNVs with approximately 3000 CAKUT cases found that 6.8% of patients harboring either the CNVs associated with a syndrome disorder or the novel, large, rare CNVs (Verbitsky et al. 2019). A closer look at common genomic disorder loci attributes specificity to CAKUT subcategories, such as *17q12* del with the specific phenotype of kidney anomality (Verbitsky et al. 2019). Patients with CAKUT have variable phenotypes, and the disease may occur in conjunction with defects in other organs. When a pathogenic variant is found in certain genes, screening for ocular coloboma or diabetes caused by *PAX2* or *HNF1B* respectively, should be recommended. The complex genetic basis of CAKUT calls for large-scale genetic studies accompanied by deep phenotyping.

We also presented the spectrum of pathogenic genes in each disease subgroup which focused on the allelic distributions in different populations. Among podocytopathies, for example, the most frequently mutated genes were *WT1, COQ8B* and *NPHS1*, rather than *NPHS1, WT1* and *NPHS2* reported by other groups mostly involving the Caucasian population (Warejko et al. 2018). In renal calcinosis and stone, the most frequently mutated genes found in the Chinese pediatric cohort were *AGXT*, *OGA1,* and *SLC3A1*, which is quite different from the genetic spectrum of *SLC7A9, SLC3A1,* and *ADCY10* reported in Caucasian individuals (Kleta and Bockenhauer 2018; Halbritter et al. 2018). We also found the most common pathogenic genes, *NPHP1* and *NPHP3*, which were carried by 52% of Chinese children with NPHP-related ciliopathies.

Phenocopies of monogenic nephropathy may occur more frequently than expected. ES for an extended virtual panel of nephropathy-related genes and reverse phenotyping may help to identify the phenocopies. A phenocopy is defined as “a phenotypic trait or disease that resembles the trait expressed by a particular genotype, but in an individual who is not a carrier of that genotype” (Pollak and Friedman 2020). Sources of phenotypic complexity in nephropathies include pleiotropy, incomplete penetrance, and variable expressivity (Groopman et al. 2020). By adding a further step of reverse phenotyping, we increased the rate of rescued diagnoses to 1.8% owing to the identification of other genetic disorders appearing as phenocopies of monogenic nephropathies. This demonstrated the power of reverse phenotyping for the interpretation of sequencing data directly in the clinical setting. For certain genetic diseases, syndromic features may be nonpenetrant in the patients as well as in the families, or may become evident only with age. In these patients, extrarenal involvement and syndromic features may be subtle and need to be specifically assessed to reach conclusive diagnoses. Therefore, we suggest that for the phenocopy genes, reverse phenotyping of the patients and relatives should always be a part of the diagnostic workup with geneticists, nephrologists, and nephropathologists to avoid misdiagnosis. The precise definition of a phenotype may assist in genetic detection, and genetic findings may lead to a refined understanding of phenotypic variability (Pollak and Friedman 2020). A group of Chinese pediatricians and geneticists from the “Internet Plus” Nephrology Alliance of the National Center for Children’s Care implemented a combined analysis of phenotype and genotype testing in a registry-based study. Identification of phenocopies helps to avoid unnecessary multiple immunosuppressive treatments and helps to decide a treatment, particularly when a special therapy is available (e.g., *COQ8B* nephropathy with CoQ10 supplementation). In addition, correct identification of phenocopies will allow to accurately enroll the patients in clinical trials and establish their prognosis on a case-by-case basis, determined by the underlying disorder (e.g. Fabry disease).

There were some limitations to this study. First, our cohort was not a population-based cohort of Chinese children. There may be a selection of patients with more severe situations sent to the medical centers enrolled in the network. However, it is the largest Chinese pediatric renal disease cohort reported to date. Based on the geographic distribution of the 51 medical centers in 23 provinces, it suggests that our findings are relevant to other parts of China or elsewhere. The differences in the geographical distribution of pediatric patients with kidney disease reported in our study may be due to unbalanced regional development in screening for CKD and in the genetic diagnosis of kidney disease. Irrespective of several cases with a delayed diagnosis because of unbalanced regional development in prenatal ultrasound screening for CAKUT or neglecting the extrarenal phenotype, the spectrum of phenotype and genotype shown in our study provided clinical clues to suspected genetic kidney disease. Second, we did not find a significant difference in the diagnostic yield in patients with extrarenal features compared to the patients without extrarenal features. In addition, only 6% (138/2,256) of patients reported the extrarenal phenotype, which was much lower than that reported in other cohorts of genetic kidney disease (14%–53%) (Connaughton et al. 2019; Groopman et al. 2019). No dual molecular diagnoses were identified in this study. There could be many cases with the unrecognized extrarenal phenotypes. We are going to contact physicians to perform detailed investigations on the extrarenal phenotypes and follow-up information, especially for the unsolved cases. Third, we did not find an association between age at initial presentation and a positive diagnosis. A lower genetic diagnostic yield was associated with a higher proband age in some studies when the diagnostic ratio in the adult cohort was compared to that in the pediatric cohort (Vivante and Hildebrandt 2016; Sadowski et al. 2015). Other studies confirmed a similar overall diagnostic rate between adults and children (Connaughton et al. 2019; Mallett et al. 2017). A recent study showed that age at first presentation of renal disease was an independent clinical predictor of genetic diagnosis on multivariable analysis; however, diagnostic ratios between age group of less than 1 year old and 1–17 years old were not significantly different. The dissimilarities in diagnostic yield between these studies may result from the differences in sample size, inclusion criteria, sequencing approaches, and healthcare regions for the clinical early detection of kidney diseases. Further analysis of the clinical impact of genomic diagnosis in different age groups should be performed. Fourth, carriers of genetic kidney disease without proteinuria or clinical symptoms could be missed according to the criterion of enrollment. For example, female carriers of Alport syndrome who presented with only microscopic hematuria may not be included in our study if no family history of kidney disease was provided. A program for genetic carrier screening for kidney diseases will be performed in the future.

## Conclusion

Combined analysis of the phenotype and the genotype reveals the underlying genetic causes in a significant proportion of pediatric patients with kidney and urological disease. A precise definition of a phenotype may assist in the genetic diagnosis. Genetic findings can contribute to a better understanding of the phenotypic variability in kidney diseases.

## Supplementary Information

Below is the link to the electronic supplementary material.

Supplementary file1 (PDF 1592 kb)

## Data Availability

The data that support the findings of this study are available from the corresponding author upon reasonable request. Not applicable.
