# Supplementary material for: Genetic Architecture of Childhood Kidney and Urological Diseases in China
Source: Phenomics. 2021 Jul 15;1(3):91–104. doi: 10.1007/s43657-021-00014-1 (PMC9590557; doi:10.1007/s43657-021-00014-1)
Supplement: Supplementary file 1 — Supplementary file1 (PDF 1592 kb) [file 43657_2021_14_MOESM1_ESM.pdf]

SUPPLEMENTARY INFORMATION
Genetic architecture of childhood kidney and urological diseases in China

Ye FANG[1,2,3*], Hua SHI[1,2,3*], Tianchao XIANG[1,2,3*], Jiaojiao LIU[1,2,3], Jialu LIU[1,2,3], Xiaoshan TANG[1,2,3], Xiaoyan FANG[1,2,3], Jing CHEN[1,2,3], Yihui ZHAI[1,2,3], Qian SHEN[1,2,3], Guomin LI[4], Li SUN[4], Yunli BI[5], Xiang WANG[5], Yanyan QIAN[6], Bingbing WU[6], Huijun WANG[6], Wenhao ZHOU[6], Duan MA[3,7], Jianhua MAO[8], Xiaoyun JIANG[9],Shuzhen SUN[10], Ying SHEN[11], Xiao rong LIU[11], Aihua ZHANG[12], Xiaowen WANG[13], Wenyan HUANG[14], Qiu LI[15], Mo WANG[15], Xiaojie GAO[16], Yubin WU[17], Fang DENG[18], Ruifeng ZHANG[19], Cuihua LIU[20], Li YU[21], Jieqiu ZHUANG[22], Qing SUN[23], Xiqiang DANG[24], Haitao BAI[25], Ying ZHU[26], Siguang LU[27], Bili ZHANG[28], Xiaoshan SHAO[29], Xuemei LIU[30], Mei HAN[31], Lijun ZHAO[32], Yuling LIU[33], Jian GAO[34], Ying BAO[35], Dongfeng ZHANG[36], Qingshan MA[37], Liping ZHAO[38], Zhengkun XIA[39], Biao LU[40], Yulong WANG[41], Mengzhun ZHAO[42], Jianjiang ZHANG[43], Shan JIAN[44], Guohua HE[45], Huifeng ZHANG[46], Bo ZHAO[47], Xiaohua LI[48], Feiyan WANG[49], Yufeng LI[50], Hongtao ZHU[51], Xinhui LUO[52], Jinghai LI[53], Jia RAO[1,2,3], Hong XU[1,2,3]

# Correspondence should be addressed to:

Hong XU, M.D., Ph.D.

Department of Nephrology, Children's Hospital of Fudan University,

National Pediatric Medical Center of CHINA,

399 Wanyuan Road, Shanghai, China

Phone: 86-21-64931006

Email: hxu@shmu.edu.cn

Jia RAO, M.D., Ph.D.

Department of Nephrology, Children's Hospital of Fudan University,

National Pediatric Medical Center of CHINA,

399 Wanyuan Road, Shanghai, China

Phone: 86-21-64932881

Email: jiarao@fudan.edu.cn

Online resource 1. Leading medical centers enrolled in Chinese Children Genetic Kidney Disease Database (CCGKDD)

Online resource 2. Supplementary Methods

Online resource 3. The patients enrollment from the subgroups of different primary diagnosis.

Online resource 4. Identifying mutations in 883 patients post WES study.

Online resource 5. Percentage of complete data with its breakdown and incomplete data

Online resource 1. Leading medical centers enrolled in Chinese Children Genetic Kidney Disease Database (CCGKDD)

Medical centers	Cases
Children's Hospital of Fudan University, Shanghai, China	1038
The Children Hospital of Zhejiang University School of Medicine, Hangzhou, China	147
The First Affiliated Hospital of Sun Yat-sen University, Guangzhou, China	140
Shandong Provincial Hospital,Jinan,China	119
Bejing Children's Hospital Affiliated to Capital University of Medical Science, Beijing, China	116
Bejing Children's Hospital Affiliated to Capital University of Medical Science, Beijing, China	116
Children's Hospital of Nanjing Medical University, Nanjing, China	114
Wuhan Children's Hospital, Tongji Medical College, Huazhong University of Science & Technology, Wuhan, China	110
Shanghai Children's Medical Centre, Shanghai Jiaotong University School of Medicine, Shanghai, China.	73
Children's Hospital of Chongqing Medical University,Chongqing,China	57
Children's Hospital of Chongqing Medical University,Chongqing,China	57
Shenzhen Children's Hospital,Shenzheng,China	52
Shengfing Hospital of China Medical University,Shenyang,Liaoning,China	35
Anhui Provincial Children's Hospital,Hefei,China	30
Xuzhou Children's Hospital,Xuzhou,China	28
Henan Children's Hospital,Zhenzhou,China	27
Guangzhou First People's Hospital,Guangzhou,China	19
The Second Affiliated Hospital and Yuying Children's Hospital of Wenzhou Medical University,Wenzhou,China	18
Qingdao Women and Children's Hospital,Qingdao,China	14
Xiangya Hospital Central South University, Changsha, Hunan, China.	14
The First Affiliated Hospital of Xiamen University, Xiamen,China	12
First Affiliated Hospital of Anhui Medical University,Hefei,China	10
Children's Hospital of Lianyungang,Lianyungang,China	8
Tianjin Children Hospital,Tianjing.China	8
The Children's Hospital of Guiyang City, Guiyang,China	7
Qilu Children's Hospital of Shandong University,Jinan,China	7
DaLian Children's Hospital, Dalian, China	6
Shanxi Children's Hospital,Taiyuan,China	6
Boai Hospital of Zhongshan,Zhongshan,China	4
Weifang Maternal and Child Health Hospital,Weifang,China	4
Xi'an Children's Hospital,Xian,China	4
Children's Hospital of Hebei Province,Shijiazhuang,China	3
First Affiliated Hospital of Jilin University,Changchun,China	3
Wuxi Municipal Children's Hospital,Wuxi,China	3
Department of Pediatrics, Jinling Hospital, Nanjing Medical University, Nanjing, 210002, China	2
General Hospital of Ningxia Medical University,Yingchuan,China	2
The Second Hospital of Shandong University,Jinan,China	2
Shenzhen Hospital of University of Hong Kong,Shenzhen,China	2
First Affiliated Hospital of Zhengzhou University,Zhengzhou,China	2
Peking Union Medical College Hospital,Beijing,China	1
Child Health Hospital of Foshan, Foshan, Guangdong, China.	1
The Second Hospital of Hebei Medical University,Shijiazhuang,China	1
Kunming Children's Hospital,Kunming,China	1
Affiliated Hospital of Inner Mongolia Medical University,Hohehot,China	1
Urumqi City Children's Hospital, Urumqi, China	1
Xinhua Hospital Affiliated to Medical College of Shanghai Jiaotong University,Shanghai,China	1
Academy of Pediatrics, Xinjiang Medical University,Urumqi, China	1
Xinjiang Uygur Autonomous Region People's Hospital, Urumqi, China	1
Changchun Children's Hospital,Changchun,China	1

Online resource 2. Supplementary Methods

Whole-exome sequencing

All patients and their unaffected family members selected underwent WES for diagnosing kidney disease. Genomic DNA was isolated from blood lymphocytes and was fragmented to an average size of 250 bp. End repair, adapter ligation, and PCR enrichment were performed following the protocol for VAHTS TM Universal DNA Library Prep Kit for Illumina V3 (Vazyme Biotech Co., Ltd, Nanjing, China). The enriched DNA libraries were subjected to exome capture using Agilent SureSelect Clinical Research Exome V2 or Human All Exon V7. The resulting libraries were sequenced on Illumina sequencers (HiSeq 4000 or Hiseq X) with the paired-end of 150 bp at Precision Medicine Center of Zhengzhou Univerisity.

Bioinformatics processing

Sequencing adapters and low-quality reads were trimmed from raw reads with Trimmomatic 1. Clean reads were then mapped to the human reference genome assembly (GRCh37) using Burrow-Wheeler Aligner (version 0.7.17-r1188) 2. Single nucleotide variants (SNVs) and small indels were characterized using the HaplotypeCaller from the Genome Analysis Toolkit (version 4, GATK4) 3. Variants were annotated using SnpEff 4 and Vcfanno 5 with a number of databases for predicted effect on protein function, allele frequency in healthy population (1000 Genomes Project database 6, dbSNP 7, Exome Aggregation Consortium ExAC 8, and Genome Aggregation Database gnomAD 9), pathogenicity annotations (ClinVar 10, InterVar 11 and HGMD 12), and for *in silico* pathogenicity predictions in dbNSFP 13, which compiled mutation prediction scores from many algorithms. All analysis steps described above were performed in the framework of bcbio-nextgen (https://github.com/bcbio/bcbio-nextgen).

Key resources used for bioinformatics processing are listed below:

**Resource**	**URL**
SnpEff	http://snpeff.sourceforge.net/
Vcfanno	https://github.com/brentp/vcfanno/
1000 Genomes Project	http://www.internationalgenome.org/

dbSNP	https://www.ncbi.nlm.nih.gov/SNP/
ExAC	http://exac.broadinstitute.org/
gnomAD	http://gnomad.broadinstitute.org/
ClinVar	https://www.ncbi.nlm.nih.gov/clinvar/
InterVar	http://wintervar.wglab.org/
HGMD	http://www.hgmd.cf.ac.uk/ac/index.php
dbNSFP	https://sites.google.com/site/jpopgen/dbNSFP

Variant filtering and interpretation

The identified variants with SnpEff annotation effects including intergenic_region, upstream_gene_variant, downstream_gene_variant, 5_prime_UTR_variant, 3_prime_UTR_variant, intron_variant, and non_coding_transcript_exon_variant were further removed. At the same time, variants with ada_score >0.5, or rf_score >0.5, or were annotated as likely pathogenic or pathogenic or VUS in ClinVar database were kept. We then filtered out variants with minor allele frequency >0.05 in any general continental population in which at least 2,000 alleles were observed in the gnomAD database, except those on ACMG benign stand-alone exception list [14].

The resulting variant set was loaded into the GEMINI [15] for exploring variants based on inheritance patterns, including autosomal dominant, *de novo*, autosomal recessive, compound heterozygous, X-linked dominant, and X-linked recessive. We then prioritized variants that occurred in the nephropathy associated genes list (Box 1). Variant interpretation (Box 2) was performed by a panel of an nephrologist with expertise in inherited kidney diseases, a bioinformatician, and a molecular geneticist using the American College of Medical Genetics and Genomics and the Association for Molecular Pathology (ACMG) guidelines for clinical sequence interpretation [16]. Diagnostic variants were defined as "pathogenic" or "likely pathogenic" according ACMG guidelines. And we also evaluate the variants of uncertain significance (VUS) of known disease causative genes through combined analysis with genotype and phenotype. All diagnostic variants were confirmed by Sanger sequencing and tested for co-segregation in the family.

Box 1. Variant filtering strategy for identifying the potential pathogenic variants in genes known to cause kidney disease

i. Keep rare variants present with a minor allele frequency (MAF) <1% in healthy control cohorts
dbSNP147 (https://www.ncbi.nlm.nih.gov/projects/SNP).

ii. Keep non-synonymous variants and intronic variants that are located within splice sites.

iii. Applying known gene approach by selecting all variants detected in known kidney disease genes.[17]

iv. Ranking of remaining variants based on their predicted likelihood to be deleterious for the function of the encoded protein using Polyphen 2
(http://genetics.bwh.harvard.edu/pph2, SIFT (http://sift.jcvi.org/) and Mutation Taster (http://www.mutationtaster.org)

v. Reviewing literature and review with referring physician delineating whether the detected mutation matches the phenotype.

vi. Cross reference with the ACMG guidelines to determine if pathogenic, likely pathogenic or a variant of uncertain signi

Box 2. Variant analysis criteria

Autosomal recessive variants

Disease-causing variants in recessive genes were considered if two alleles were found in the same individual that fulfilled at least one of the following criteria:

i) Truncating allele (stop, abrogation of start or stop, obligatory splice site, or frameshift); OR

ii) Missense mutation if a minimum of 4 of 5 of the following criteria were met:

- Continuously conserved at least among vertebrates (or beyond)
- Previously reported as disease causing or functional evidence implicating causality
- Loss of function in human allele is supported by functional data
- Phenotype correlates with the published phenotype for the gene
- Predicted deleterious for the protein function (at least in two among three prediction programs (Polyphen (>0.5), SIFT (Del.), Mutation taster (D.C.))

Exclude allele as disease causing if:

- Allele frequency >1% (in ExAC, gnomAD, 1000 genomes)
- Non segregation: if compound heterozygous variants are in cis or if an affected family member is without the variant or an unaffected family member is with the variant

Discussion of genotype-phenotype correlation in a panel of nephro-geneticists followed by review of clinical phenotype with referring physician

Autosomal dominant variants

Disease-causing variants in dominant genes were considered if one allele fulfilled at least one of the following criteria:

i) Truncating mutation (stop, abrogation of start or stop, obligatory splice site, and frameshift); OR

ii) Missense mutation if a minimum of 4 of 5 of the following criteria were met:

- Continuously conserved at least among vertebrates (or beyond)
- Previously reported as disease causing or functional evidence implicating causality
- Phenotype correlates with the published phenotype for the gene
- Predicted deleterious for the protein function (at least in two among three prediction programs (Polyphen (>0.5), SIFT (Del.), Mutation taster (D.C.))

Exclusion criteria:

- Allele frequency >0.1% ((in, ExAC, gnomAD, 1000 genomes)
- Non segregation:if the allele did not segregate with the affected status in the family.

Or If an unaffected family member is with the allele consider incomplete penetrance and variable expressivity

Discussion of genotype-phenotype correlation in a panel of nephro-geneticists followed by review of clinical phenotype with referring physician

Quality control of sequencing data

The QC was performed at many stages of the analysis pipeline, including pre-cleaning, post-cleaning, post-alignment, and post-variant-calling. The average sequencing depth of targeted regions is 98.3X, with 95% on average of the targeted based sequenced at least 20 times.

Qualitative data verification

Data quality is evaluated by relevance, completeness and accuracy[18]. Quantitatively, all data in the CCGKDD (Chinese Children Genetic Kidney Disease Database, www. ccgkdd.com.cn) were entered into a computer by research assistants. Combined data entered into a single cell (e.g. dates as day/month/year) were divided so that each piece of data was entered into a separate cell. In total, 38 different data categories were analyzed (Table 2). Date, numerical data and categorical data were entered as they were, and illegible data were given a new code. Incorrectly coded and inappropriate data were identified. Inappropriate data were those that do not exist within the presumed range (e.g. ‘13’ for month). Nominal data (name and residence) and numbers (references and birth certificate) were newly coded as legible or illegible, and residence was further categorized by local research assistants as recognized or not recognized. Completeness was evaluated by counting cells filled with data. Complete data that were illegible, incorrectly coded, inappropriate or unrecognized

were counted to assess accuracy. The data categories in the registers were qualitatively compared with those in the quarterly workload reports to determine whether these categories were relevant to diagnosis and management needs. The quarterly workload reports presented to the council members of “Internet Plus” Nephrology Alliance of National Center for Children’s Care”. And the annual reports on data quality were presented on the website.

Measures of data quality verification used in this study

Quantitative verification

- Counting the total number of deliveries and comparing this with the number in a quarterly workload report (accuracy)
- Counting the complete and incomplete data (completeness)
- Counting the complete data which were illegible, wrongly coded, inappropriate and unrecognised (accuracy)

Qualitative verification

- Comparing the data categories with those in a quarterly workload report to evaluate whether the data collected satisfied management information needs (relevance)
- Examining the instructions, with the input by network management working group, to evaluate their influence to data accuracy and incompleteness
- Examining the views and factors that influence data quality of the delivery registers through key informant interviews

---

References

1. Bolger AM, Lohse M, Usadel B. Trimmomatic: a flexible trimmer for Illumina sequence data. Bioinformatics 2014;30:2114-20.
2. Li H. Aligning sequence reads, clone sequences and assembly contigs with BWA-MEM. arXiv 2013;1303.3997.
3. DePristo MA, Banks E, Poplin R, et al. A framework for variation discovery and genotyping using next-generation DNA sequencing data. Nature Genetics 2011;43:491-8.
4. Cingolani P, Platts A, Wang le L, et al. A program for annotating and predicting the effects of single nucleotide polymorphisms, SnpEff: SNPs in the genome of Drosophila melanogaster strain w1118; iso-2; iso-3. Fly (Austin) 2012;6:80-92.
5. Pedersen BS, Layer RM, Quinlan AR. Vcfanno: fast, flexible annotation of genetic variants. Genome biology 2016;17:118.
6. Genomes Project C, Auton A, Brooks LD, et al. A global reference for human genetic variation. Nature 2015;526:68-74.
7. Sherry ST, Ward MH, Kholodov M, et al. dbSNP: the NCBI database of genetic variation. Nucleic acids research 2001;29:308-11.
8. Lek M, Karczewski KJ, Minikel EV, et al. Analysis of protein-coding genetic variation in 60,706 humans. Nature 2016;536:285-91.
9. Karczewski KJ, Francioli LC, Tiao G, et al. Variation across 141,456 human exomes and genomes reveals the spectrum of loss-of-function intolerance across human protein-coding genes. bioRxiv 2019:531210.
10. Landrum MJ, Lee JM, Benson M, et al. ClinVar: improving access to variant interpretations and supporting evidence. Nucleic acids research 2018;46:D1062-D7.
11. Li Q, Wang K. InterVar: Clinical Interpretation of Genetic Variants by the 2015 ACMG-AMP Guidelines. American journal of human genetics 2017;100:267-80.
12. Stenson PD, Ball EV, Mort M, Phillips AD, Shaw K, Cooper DN. The Human Gene Mutation Database (HGMD) and its exploitation in the fields of personalized genomics and molecular evolution. Current protocols in bioinformatics 2012;Chapter 1:Unit1 13.
13. Liu X, Wu C, Li C, Boerwinkle E. dbNSFP v3.0: A One-Stop Database of Functional Predictions and Annotations for Human Nonsynonymous and Splice-Site SNVs. Human mutation 2016;37:235-41.
14. Ghosh R, Harrison SM, Rehm HL, Plon SE, Biesecker LG, ClinGen Sequence Variant Interpretation Working G. Updated recommendation for the benign stand-alone ACMG/AMP criterion. Human mutation 2018;39:1525-30.
15. Paila U, Chapman BA, Kirchner R, Quinlan AR. GEMINI: integrative exploration of genetic variation and genome annotations. PLoS computational biology 2013;9:e1003153.
16. Richards S, Aziz N, Bale S, et al. Standards and guidelines for the interpretation of sequence variants: a joint consensus recommendation of the American College of Medical Genetics and Genomics and the Association for Molecular Pathology. Genet Med 2015;17:405-24.
17. Groopman EE, Marasa M, Cameron-Christie S, et al. Diagnostic Utility of Exome Sequencing for Kidney Disease. N Engl J Med 2019;380:142-51.
18. Blobel B. Analysis, design and implementation of secure and interoperable distributed health information systems. Stud Health Technol Inform 2002;89:1-329.

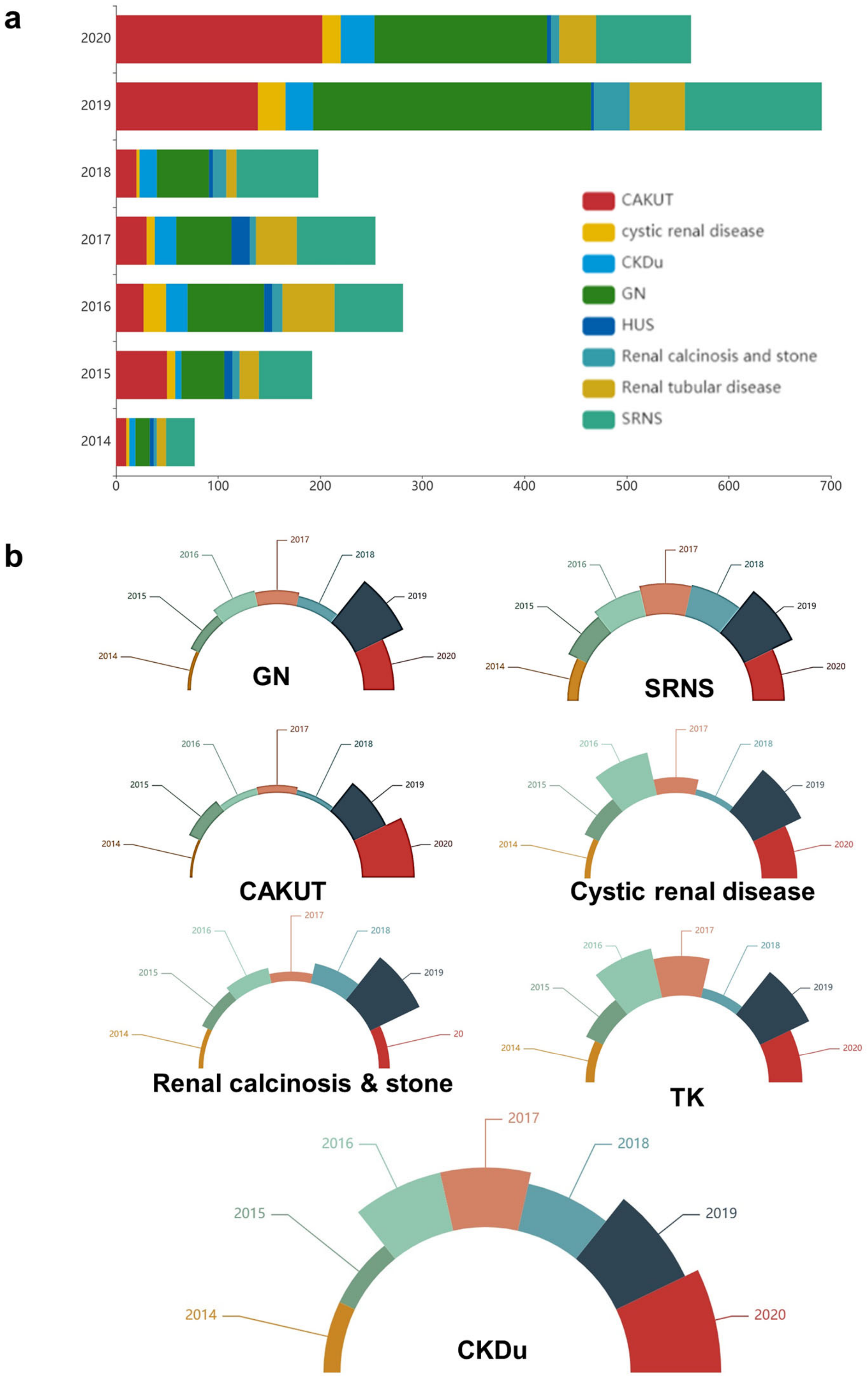

Online resource 3. The patients enrollment from the subgroups of different primary diagnosis. (a) The patient number in different subgroups of patients with different primary diagnosis enrolled from 2014 to 2020; (b) Percentage of each subgroups in different enrolled year

Online resource 4. Identifying mutations in 883 patients post WES study.

ID	Gender(M, male; F, female)	Age at genetic study (years)	Year registy	Area	Categ0ry of primary diagnosis	Gene with pathogenic variants	Diagnosis post WES
PD1111113	M	3.9	2017	Eastern Area	Renal tubular disease	*PHEX*	TID
PD11111712	M	1.0	2017	Eastern Area	HUS	*DGKE*	HUS
PD11111711	F	1.3	2017	Eastern Area	GN	*COL4A5*	Alport syn.
PD1111123	F	8.0	2017	Eastern Area	ESRD	*MMACHC*	Metabolic disorders
PD11111708	M	4.0	2017	Eastern Area	GN	*COL4A5*	Alport syn.
PD1111126	M	0.3	2017	Eastern Area	ESRD	*MMACHC*	Metabolic disorders
PD11111706	F	13.0	2017	Eastern Area	GN	*COL4A5*	Alport syn.
PD11111705	M	5.0	2017	Eastern Area	SRNS	*INF2*	FSGS
PD11111702	M	5.8	2017	Eastern Area	GN	*CFHR5*	GN
PD11111700	M	3.0	2017	Eastern Area	ESRD	*MMACHC*	Metabolic disorders
PD11111699	M	1.3	2017	Eastern Area	HUS	*CFH*	HUS
PD11111698	M	1.2	2017	Eastern Area	HUS	*CD46*	HUS
PD11111697	F	10.0	2017	Eastern Area	SRNS	*INF2*	FSGS
PD11111696	M	13.0	2017	Eastern Area	GN	*COL4A5*	Alport syn.
PD11111695	M	3.7	2017	Eastern Area	ESRD	*MMACHC*	Metabolic disorders
PD1111159	M	7.0	2017	Eastern Area	ESRD	*MMACHC*	Metabolic disorders
PD11111693	M	0.9	2017	Eastern Area	HUS	*C3*	HUS
PD11111692	F	1.0	2017	Eastern Area	CAKUT	*SALL1*	CAKUT
PD1111169	M	2.3	2017	Eastern Area	Renal tubular disease	*OCRL*	TID
PD11111690	M	1.0	2017	Eastern Area	SRNS	*WT1*	FSGS
PD1111180	M	1.0	2017	Eastern Area	Renal tubular disease	*AVPR2*	TID
PD1111187	F	5.7	2017	Eastern Area	SRNS	*ANLN*	FSGS
PD30000868	M	4.6	2016	Eastern Area	GN	*COL4A4*	Alport syn.
PD30001236	F	10.7	2016	Eastern Area	cystic renal disease	*PKD1*	cystic renal disease
PD30000889	F	2.0	2016	Eastern Area	GN	*COL4A5*	Alport syn.
PD11111898	M	13.0	2016	Eastern Area	cystic renal disease	*PKD1*	cystic renal disease
PD11111897	M	7.0	2016	Eastern Area	Renal tubular disease	*CLCN5*	TID
PD30000890	M	2.0	2016	Eastern Area	GN	*COL4A5*	Alport syn.
PD11111106	F	4.5	2016	Eastern Area	cystic renal disease	*PKD2*	cystic renal disease
PD11111111	F	5.1	2016	Eastern Area	Renal tubular disease	*SLC12A3*	TID
PD11112808	F	3.7	2016	Eastern Area	Renal tubular disease	*KCNJ5*	TID
PD11111891	M	5.0	2016	Eastern Area	Renal tubular disease	*CLCN5*	TID
PD30000870	M	12.0	2016	Eastern Area	GN	*COL4A4*	Alport syn.
PD30000887	M	5.0	2016	Eastern Area	SRNS	*ANLN*	FSGS
PD11111127	F	6.0	2016	Eastern Area	Renal tubular disease	*EHHADH*	TID
PD11111883	F	8.0	2016	Eastern Area	GN	*COL4A5*	Alport syn.
PD11111139	F	4.0	2016	Eastern Area	Renal tubular disease	*CLCN5*	TID
PD30000878	F	2.8	2016	Eastern Area	GN	*COL4A5*	Alport syn.
PD30000869	F	12.0	2016	Eastern Area	GN	*COL4A4*	Alport syn.
PD11111876	M	6.4	2016	Eastern Area	Renal tubular disease	*CLCN5*	TID
PD30000875	M	6.2	2016	Eastern Area	GN	*COL4A5*	Alport syn.
PD11111157	M	3.0	2016	Eastern Area	cystic renal disease	*PKD1*	cystic renal disease
PD30000781	F	5.0	2016	Eastern Area	SRNS	*INF2*	FSGS
PD30000871	M	7.0	2016	Eastern Area	GN	*COL4A4*	Alport syn.
PD30000877	M	12.0	2016	Eastern Area	GN	*COL4A5*	Alport syn.
PD11111864	F	1.6	2016	Eastern Area	Renal tubular disease	*SLC4A1*	TID
PD30000876	M	6.0	2016	Eastern Area	GN	*COL4A5*	Alport syn.
PD30000874	F	5.6	2016	Eastern Area	GN	*COL4A5*	Alport syn.
PD11111861	F	0.9	2016	Eastern Area	HUS	*CFH*	HUS
PD30000885	M	7.0	2016	Eastern Area	GN	*NPHS2*	FSGS
PD30000888	F	12.0	2016	Eastern Area	GN	*COL4A5*	Alport syn.
PD30000883	M	11.0	2016	Eastern Area	CAKUT	*PAX2*	CAKUT
PD11111856	M	2.5	2016	Eastern Area	CAKUT	*ROBO2*	CAKUT
PD11111851	M	8.9	2016	Eastern Area	GN	*COL4A3*	Alport syn.
PD30000078	F	1.6	2016	Eastern Area	GN	*COL4A5*	Alport syn.
PD30000076	M	9.0	2016	Eastern Area	GN	*COL4A5*	Alport syn.
PD30000075	F	4.3	2016	Eastern Area	SRNS	*TTC21B*	FSGS
PD30000072	M	12.8	2016	Eastern Area	CAKUT	*HNF1B*	CAKUT
PD30000083	F	4.0	2016	Eastern Area	Renal tubular disease	*ATP6V1B1*	TID
PD30000077	M	2.0	2016	Eastern Area	Renal tubular disease	*OCRL*	TID
PD30000079	M	7.0	2016	Eastern Area	Renal tubular disease	*SLC12A3*	TID
PD11111730	M	0.8	2016	Eastern Area	Renal tubular disease	*CLCN5*	TID
PD11111729	M	9.0	2016	Eastern Area	Renal tubular disease	*OCRL*	TID
PD30000065	M	3.4	2016	Eastern Area	GN	*COL4A3*	Alport syn.
PD11111672	F	4.0	2015	Middle Region	GN	*COL4A5*	Alport syn.
PD11111219	M	0.7	2015	Middle Region	Renal tubular disease	*SLC4A1*	TID
PD11111790	M	10.6	2015	Middle Region	GN	*COL4A3*	Alport syn.
PD11111789	F	2.8	2015	Middle Region	Renal tubular disease	*PHEX*	TID
PD11111788	F	3.0	2015	Middle Region	HUS	*CFH*	HUS
PD11111787	F	8.0	2015	Middle Region	SRNS	*COL4A3*	FSGS
PD11111785	M	2.8	2015	Middle Region	GN	*COL4A4*	Alport syn.
PD11111792	M	2.0	2015	Middle Region	GN	*COL4A5*	Alport syn.
PD11111784	M	8.0	2015	Middle Region	GN	*COL4A4*	Alport syn.
PD11111238	M	8.0	2015	Middle Region	SRNS	*ADCK4*	FSGS
PD11111782	M	11.0	2015	Middle Region	SRNS	*INF2*	FSGS
PD11111781	M	5.0	2015	Middle Region	GN	*COL4A5*	Alport syn.
PD11112599	F	2.3	2015	Middle Region	SRNS	*COL4A5*	Alport syn.
PD11111780	M	1.7	2015	Middle Region	GN	*COL4A5*	Alport syn.
PD11111245	F	3.3	2015	Middle Region	GN	*COL4A3*	Alport syn.
PD11111669	F	3.1	2015	Middle Region	SRNS	*COL4A5*	Alport syn.
PD11111778	F	5.7	2015	Middle Region	SRNS	*COL4A3*	FSGS
PD11111773	M	7.9	2015	Middle Region	cystic renal disease	*PKHD1*	cystic renal disease
PD11111671	M	12.0	2015	Middle Region	GN	*COL4A5*	Alport syn.
PD11111256	M	4.7	2015	Middle Region	GN	*COL4A5*	Alport syn.
PD11111257	M	6.4	2015	Middle Region	GN	*COL4A3*	Alport syn.
PD11111769	M	1.5	2015	Middle Region	SRNS	*WT1*	FSGS

PD11111768	F	0.8	2015	Middle Region	Renal calcinosis and stone	*SLC3A1*	Renal calcinosis and stone
PD30001592	M	13.0	2015	Western Area	GN	*COL4A5*	Alport syn.
PD11111919	F	9.0	2016	Western Area	GN	*COL4A5*	Alport syn.
PD11111918	F	2.8	2015	Western Area	Renal tubular disease	*SLC4A1*	TID
PD11111917	M	2.1	2016	Western Area	Renal tubular disease	*CASR*	TID
PD11111913	M	10.0	2015	Western Area	cystic renal disease	*PKD1*	cystic renal disease
PD11111279	M	3.3	2015	Western Area	Renal tubular disease	*OCRL*	TID
PD11111280	F	3.7	2016	Western Area	GN	*COL4A5*	Alport syn.
PD11111285	F	0.1	2016	Western Area	cystic renal disease	*PKHD1*	cystic renal disease
PD11111286	M	8.0	2015	Western Area	GN	*COL4A4*	Alport syn.
PD11111664	M	13.0	2016	Middle Region	GN	*COL4A5*	Alport syn.
PD11111927	M	3.0	2016	Middle Region	GN	*COL4A5*	Alport syn.
PD11111298	F	0.4	2016	Middle Region	CAKUT	*ACTG2*	CAKUT
PD11111301	M	2.0	2015	Eastern Area	CKDU	*OCRL*	TID
PD11111306	M	13.0	2015	Eastern Area	Renal tubular disease	*SCNN1G*	TID
PD11111754	M	12.0	2016	Eastern Area	GN	*COL4A5*	Alport syn.
PD30002114	M	8.0	2015	Eastern Area	SRNS	*COL4A3*	FSGS
PD30000251	M	1.3	2016	Eastern Area	SRNS	*OCRL*	FSGS
PD11111755	M	8.0	2016	Eastern Area	GN	*COL4A4*	Alport syn.
PD11111322	F	0.3	2015	Eastern Area	Renal tubular disease	*SLC34A3*	TID
PD11111324	F	11.0	2017	Eastern Area	SRNS	*ADCK4*	FSGS
PD11111758	M	1.0	2016	Eastern Area	CKDU	*NPHP3*	NPHP
PD11111334	M	4.0	2017	Eastern Area	HUS	*CFH*	HUS
PD11111335	M	16.0	2015	Eastern Area	Renal tubular disease	*SLC12A3*	TID
PD11111336	M	11.0	2014	Eastern Area	SRNS	*COL4A4*	FSGS
PD11111759	M	1.5	2016	Eastern Area	SRNS	*TRPC6*	FSGS
PD11111339	M	6.0	2014	Eastern Area	SRNS	*COL4A5*	Alport syn.
PD11111744	F	18.0	2016	Eastern Area	cystic renal disease	*BBS2*	cystic renal disease
PD11111537	M	5.4	2015	Eastern Area	Renal tubular disease	*CLCN5*	TID
PD11111347	F	9.0	2015	Eastern Area	SRNS	*ADCK4*	FSGS
PD30001973	F	1.0	2015	Eastern Area	cystic renal disease	*PKHD1*	cystic renal disease
PD11111762	M	2.0	2016	Eastern Area	Renal tubular disease	*UMOD*	TID
PD11111352	M	6.0	2014	Eastern Area	Renal tubular disease	*CLCN5*	TID
PD11111354	M	12.0	2014	Eastern Area	GN	*COL4A5*	Alport syn.
PD11111360	F	10.0	2015	Eastern Area	CAKUT	*ROBO2*	CAKUT
PD11111849	M	14.0	2015	Eastern Area	Renal calcinosis and stone	*HOGA1*	Renal calcinosis and stone
PD11111364	F	11.0	2015	Eastern Area	Renal tubular disease	*SCNN1B*	TID
PD11111366	F	9.3	2015	Eastern Area	SRNS	*MYH9*	FSGS
PD11111765	M	3.8	2016	Eastern Area	CKDU	*BBS12*	NPHP
PD11111808	F	11.0	2016	Eastern Area	CKDU	*NPHP14*	NPHP
PD11111372	M	8.0	2016	Eastern Area	HUS	*CFHR1*	HUS
PD11111809	M	10.0	2016	Eastern Area	GN	*COL4A5*	Alport syn.
PD11111810	M	7.0	2016	Eastern Area	GN	*COL4A5*	Alport syn.
PD11111812	M	8.0	2016	Eastern Area	SRNS	*WT1*	FSGS
PD11111392	F	1.0	2017	Eastern Area	SRNS	*NUP92*	FSGS
PD11111813	M	9.0	2016	Eastern Area	Renal tubular disease	*SLC12A1*	TID
PD11111396	M	1.0	2015	Eastern Area	GN	*COL4A3*	Alport syn.
PD11111398	M	4.0	2015	Eastern Area	CAKUT	*EYA1*	CAKUT
PD11111399	F	7.5	2014	Eastern Area	GN	*COL4A3*	Alport syn.
PD11111411	M	1.5	2015	Eastern Area	GN	*COL4A5*	Alport syn.
PD11111412	F	3.0	2015	Eastern Area	GN	*COL4A3*	Alport syn.
PD11111564	M	0.5	2015	Eastern Area	HUS	*CFH*	HUS
PD11111817	M	6.0	2015	Eastern Area	cystic renal disease	*WDR19*	cystic renal disease
PD11111818	F	6.0	2017	Eastern Area	GN	*COL4A5*	Alport syn.
PD11111428	F	3.0	2014	Eastern Area	SRNS	*PLCE1*	FSGS
PD11111820	M	0.5	2016	Eastern Area	cystic renal disease	*PKHD1*	cystic renal disease
PD11111821	M	0.3	2016	Eastern Area	Renal tubular disease	*ATP6V1B1*	TID
PD11111438	M	6.0	2014	Eastern Area	HUS	*C5*	HUS
PD11111847	M	5.0	2015	Eastern Area	Renal calcinosis and stone	*SLC3A1*	Renal calcinosis and stone
PD11111442	M	1.0	2015	Eastern Area	cystic renal disease	*PKHD1*	cystic renal disease
PD11111444	M	4.0	2015	Eastern Area	GN	*COL4A5*	Alport syn.
PD11111580	F	3.0	2016	Eastern Area	SRNS	*ADCK4*	FSGS
PD11111824	F	9.0	2016	Eastern Area	SRNS	*WT1*	FSGS
PD11111450	F	2.0	2015	Eastern Area	cystic renal disease	*PKHD1*	cystic renal disease
PD11111451	M	2.0	2015	Eastern Area	SRNS	*COL4A5*	Alport syn.
PD11111549	M	2.0	2015	Eastern Area	Renal tubular disease	*CLCN5*	TID
PD11111550	M	5.0	2016	Eastern Area	Renal tubular disease	*CLCN5*	TID
PD11111825	F	5.0	2016	Eastern Area	cystic renal disease	*PKHD1*	cystic renal disease
PD11111826	M	12.0	2016	Eastern Area	CKDU	*NPHP1*	NPHP
PD11111463	M	8.0	2017	Eastern Area	GN	*COL4A5*	Alport syn.
PD11111477	F	1.8	2015	Eastern Area	HUS	*CHF*	HUS
PD11111484	F	11.0	2014	Eastern Area	SRNS	*WT1*	FSGS
PD11111848	M	1.9	2015	Eastern Area	Renal calcinosis and stone	*GRHPR*	Renal calcinosis and stone
PD11111492	M	1.4	2014	Eastern Area	SRNS	*CD2AP*	FSGS
PD11111495	M	9.0	2015	Eastern Area	Renal tubular disease	*APOE*	TID
PD11111828	F	10.0	2016	Eastern Area	GN	*COL4A5*	Alport syn.
PD11111498	M	0.8	2015	Eastern Area	SRNS	*WT1*	FSGS
PD11111499	M	5.0	2016	Eastern Area	GN	*COL4A4*	Alport syn.
PD11111500	F	5.0	2016	Eastern Area	Renal tubular disease	*CLCNKB*	TID
PD11111505	M	2.0	2015	Eastern Area	CAKUT	*DSTYK*	CAKUT
PD11111830	M	11.0	2016	Eastern Area	SRNS	*CLCN5*	FSGS
PD11111831	M	0.7	2016	Eastern Area	SRNS	*NPHS1*	FSGS
PD11111535	M	5.0	2015	Eastern Area	HUS	*ADAMTS13*	HUS
PD11111833	M	10.0	2017	Eastern Area	GN	*OCRL*	GN
PD11111520	F	4.0	2017	Eastern Area	cystic renal disease	*PKHD1*	cystic renal disease
PD11111836	M	6.0	2016	Eastern Area	GN	*COL4A3*	Alport syn.
PD11111525	M	2.0	2014	Eastern Area	cystic renal disease	*PKHD1*	cystic renal disease
PD11111529	M	10.0	2014	Eastern Area	CKDU	*UMOD*	TID
PD11111837	M	7.0	2016	Eastern Area	SRNS	*TRPC6*	FSGS
PD11111838	F	1.8	2015	Eastern Area	GN	*COL4A5*	Alport syn.
PD11111839	M	9.0	2016	Eastern Area	CAKUT	*TNXB*	CAKUT
PD11111536	M	9.0	2016	Eastern Area	HUS	*CFHR1*	HUS
PD11111840	M	11.0	2016	Eastern Area	Renal tubular disease	*CLCNKA*	TID
PD11111841	M	6.0	2016	Eastern Area	GN	*COL4A5*	Alport syn.

PD11111569	F	9.0	2015	Eastern Area	SRNS	*ADCK4*	FSGS
PD11111546	F	7.0	2015	Eastern Area	GN	*COQ2*	FSGS
PD11111552	M	13.0	2015	Eastern Area	SRNS	*NPHS1*	FSGS
PD11111553	F	6.0	2016	Eastern Area	Renal tubular disease	*SLC12A3*	TID
PD11111554	M	7.0	2016	Eastern Area	Renal tubular disease	*SLC4A1*	TID
PD11111555	M	5.0	2016	Eastern Area	SRNS	*ADCK4*	FSGS
PD11111558	F	14.0	2016	Eastern Area	SRNS	*ADCK4*	FSGS
PD11111562	M	2.0	2015	Eastern Area	GN	*COL4A5*	Alport syn.
PD11111563	M	0.1	2014	Eastern Area	SRNS	*NPHS1*	FSGS
PD11111844	F	3.0	2016	Eastern Area	Renal tubular disease	*ATP6V0A4*	TID
PD11111566	M	5.0	2015	Eastern Area	GN	*OCRL*	GN
PD30002112	M	7.5	2015	Eastern Area	SRNS	*COL4A5*	Alport syn.
PD30002115	F	9.0	2015	Eastern Area	SRNS	*COL4A3*	FSGS
PD11111581	F	4.0	2015	Eastern Area	GN	*COL4A5*	Alport syn.
PD11111582	F	4.0	2015	Eastern Area	GN	*COL4A5*	Alport syn.
PD11111584	M	0.3	2014	Eastern Area	Renal tubular disease	*AGXT*	TID
PD11111568	M	5.0	2014	Eastern Area	HUS	*CFHR1*	HUS
PD11111586	F	1.8	2015	Eastern Area	SRNS	*PLCE1*	FSGS
PD11111589	M	6.7	2014	Eastern Area	SRNS	*PLCE1*	FSGS
PD11111591	M	0.8	2016	Eastern Area	GN	*COL4A5*	Alport syn.
PD11111595	M	4.0	2014	Eastern Area	GN	*COL4A5*	Alport syn.
PD11111556	M	5.5	2016	Eastern Area	SRNS	*CLCN5*	FSGS
PD11112024	M	5.0	2014	Western Area	cystic renal disease	*RPGRIP1L*	cystic renal disease
PD11112025	F	2.5	2016	Western Area	SRNS	*NPHS2*	FSGS
PD11112032	M	7.1	2016	Western Area	GN	*LMX1B*	FSGS
PD11112036	M	6.1	2015	Western Area	GN	*COL4A5*	Alport syn.
PD11112028	M	1.7	2017	Western Area	Renal tubular disease	*CLCN5*	TID
PD11112092	M	0.8	2017	Western Area	SRNS	*ADCK4*	FSGS
PD11112118	M	2.5	2014	Eastern Area	Renal tubular disease	*CLCN5*	TID
PD11112119	M	2.5	2016	Eastern Area	Renal tubular disease	*CLCN5*	TID
PD11112120	M	7.3	2017	Eastern Area	Renal tubular disease	*CLCN5*	TID
PD11112135	M	10.0	2017	Middle Region	GN	*COL4A5*	Alport syn.
PD11112182	M	3.0	2016	Eastern Area	CKDU	*NPHP13*	NPHP
PD11112219	F	12.5	2017	Middle Region	CKDU	*NPHP1*	NPHP
PD11112260	F	11.0	2017	Eastern Area	SRNS	*COL4A5*	Alport syn.
PD11112272	M	5.0	2017	Eastern Area	GN	*COL4A5*	Alport syn.
PD11112273	M	3.0	2017	Eastern Area	Renal tubular disease	*CLCN5*	TID
PD11112275	F	8.0	2017	Eastern Area	GN	*COL4A5*	Alport syn.
PD11112278	M	8.0	2017	Eastern Area	SRNS	*COL4A5*	Alport syn.
PD11112279	F	4.0	2017	Eastern Area	GN	*COL4A5*	Alport syn.
PD11112299	M	12.5	2017	Middle Region	CKDU	*NPHP1*	NPHP
PD11112305	M	3.6	2017	North-Eastern Ar	Renal tubular disease	*CLCN5*	TID
PD11112306	M	13.9	2016	North-Eastern Ar	SRNS	*WT1*	FSGS
PD11112308	M	5.3	2017	North-Eastern Ar	GN	*COL4A5*	Alport syn.
PD11112309	M	5.3	2016	North-Eastern Ar	Renal tubular disease	*CLCN5*	TID
PD11112310	F	9.1	2015	North-Eastern Ar	cystic renal disease	*SDCCAG8*	cystic renal disease
PD11112312	F	1.5	2015	North-Eastern Ar	GN	*COL4A3*	Alport syn.
PD11112314	F	0.1	2017	North-Eastern Ar	Renal tubular disease	*ATP6V1B1*	TID
PD11112328	F	3.3	2017	North-Eastern Ar	Renal tubular disease	*CLCNKB*	TID
PD11112355	M	1.8	2017	North-Eastern Ar	Renal tubular disease	*OCRL*	TID
PD11112315	M	8.9	2015	Eastern Area	CAKUT	*PAX2*	CAKUT
PD11112317	M	13.4	2014	Eastern Area	Renal tubular disease	*SLC12A3*	TID
PD11112318	M	1.0	2015	Eastern Area	GN	*COL4A4*	Alport syn.
PD11112322	M	6.8	2014	Eastern Area	GN	*COL4A4*	Alport syn.
PD11112642	M	9.3	2016	Eastern Area	GN	*CLCN5*	FSGS
PD11112647	M	10.3	2016	Eastern Area	cystic renal disease	*PKD1*	cystic renal disease
PD11112650	M	8.8	2016	Eastern Area	SRNS	*ADCK4*	FSGS
PD11112652	F	14.7	2016	Eastern Area	CKDU	*NPHP1*	NPHP
PD11112655	M	3.5	2016	Eastern Area	CKDU	*BBS12*	NPHP
PD11112657	F	10.3	2016	Eastern Area	Renal tubular disease	*HNF4A*	TID
PD11112658	F	5.2	2016	Eastern Area	CKDU	*INVS*	NPHP
PD11112666	M	5.8	2016	Eastern Area	Renal tubular disease	*SLC12A3*	TID
PD11112668	M	11.0	2016	Eastern Area	SRNS	*ADCK4*	FSGS
PD11112670	M	5.3	2016	Eastern Area	GN	*COL4A5*	Alport syn.
PD11112672	M	4.7	2016	Eastern Area	GN	*COL4A3*	Alport syn.
PD11112673	M	3.0	2016	Eastern Area	Renal tubular disease	*OCRL*	TID
PD11112675	F	0.8	2016	Eastern Area	SRNS	*WT1*	FSGS
PD11112680	M	0.4	2017	Eastern Area	GN	*COL4A3*	Alport syn.
PD11112683	F	4.7	2017	Eastern Area	Renal tubular disease	*SLC12A3*	TID
PD11112688	M	15.3	2017	Eastern Area	CAKUT	*PAX2*	CAKUT
PD30001899	M	7.9	2017	Eastern Area	GN	*COL4A3*	Alport syn.
PD11112695	M	4.0	2017	Eastern Area	GN	*COL4A4*	Alport syn.
PD11112696	M	2.0	2017	Eastern Area	Renal tubular disease	*CLCN5*	TID
PD11112697	M	0.4	2017	Eastern Area	cystic renal disease	*PKHD1*	cystic renal disease
PD11112704	F	4.0	2017	Eastern Area	GN	*COL4A5*	Alport syn.
PD11112705	M	3.0	2017	Eastern Area	GN	*COL4A4*	Alport syn.
PD11112708	M	7.8	2017	Eastern Area	GN	*CLCN5*	FSGS
PD11112709	M	9.0	2017	Eastern Area	GN	*COL4A5*	Alport syn.
PD11112710	M	12.0	2017	Eastern Area	Renal tubular disease	*CLCN5*	TID
PD11112712	F	7.0	2017	Eastern Area	cystic renal disease	*PKHD1*	cystic renal disease
PD11112714	M	5.0	2017	Eastern Area	Renal tubular disease	*PHEX*	TID
PD11112717	M	0.5	2017	Eastern Area	CAKUT	*HNF1B*	CAKUT
PD11112111	M	0.6	2017	Eastern Area	Renal tubular disease	*TRPM6*	TID
PD11112724	M	3.0	2017	Eastern Area	GN	*CUBN*	FSGS
PD11112116	M	4.0	2017	Eastern Area	GN	*COL4A4*	Alport syn.
PD11112727	F	9.0	2017	Eastern Area	Renal tubular disease	*SLC5A2*	TID
PD11111718	F	11.0	2017	Eastern Area	SRNS	*NPHS1*	FSGS
PD11112240	M	0.2	2017	Eastern Area	SRNS	*NPHS1*	FSGS
PD11112242	M	9.0	2017	Eastern Area	CKDU	*NPHP3*	NPHP
PD11112742	F	6.0	2017	Eastern Area	GN	*COL4A5*	Alport syn.
PD11112089	M	5.0	2017	Eastern Area	SRNS	*ADCK4*	FSGS
PD11112035	M	7.0	2017	Eastern Area	Renal calcinosis and stone	*AGXT*	Renal calcinosis and stone
PD11112750	M	11.0	2017	Eastern Area	GN	*MMACHC*	Metabolic disorders
PD11112760	F	2.0	2017	Eastern Area	cystic renal disease	*PKHD1*	cystic renal disease

PD30001497	F	13.0	2017	Eastern Area	CAKUT	*PAX2*	CAKUT
PD11112766	M	3.0	2017	Eastern Area	Renal tubular disease	*CACNA1S*	TID
PD11112767	F	0.3	2017	Eastern Area	Renal calcinosis and stone	*AGXT*	Renal calcinosis and stone
PD11112771	M	4.3	2017	Eastern Area	cystic renal disease	*PKHD1*	cystic renal disease
PD11112775	M	7.0	2017	Eastern Area	Renal tubular disease	*CLCN5*	TID
PD11112232	M	7.0	2017	Eastern Area	CAKUT	*PBX1*	CAKUT
PD11112783	F	0.8	2015	Eastern Area	SRNS	*LAMB2*	FSGS
PD11111570	M	8.0	2015	Eastern Area	SRNS	*ADCK4*	FSGS
PD11112788	M	14.9	2015	Eastern Area	GN	*COL4A5*	Alport syn.
PD30000755	F	6.0	2018	North-Eastern Ar	GN	*COL4A4*	Alport syn.
PD11111771	F	2.4	2018	North-Eastern Ar	SRNS	*NPHS1*	FSGS
PD30000249	M	2.0	2016	Eastern Area	Renal calcinosis and stone	*SLC3A1*	Renal calcinosis and stone
PD30000240	F	5.4	2017	Eastern Area	Renal calcinosis and stone	*AGXT*	Renal calcinosis and stone
PD30000244	F	0.8	2018	Eastern Area	Renal calcinosis and stone	*GRHPR*	Renal calcinosis and stone
PD30000241	M	3.5	2018	Eastern Area	Renal calcinosis and stone	*AGXT*	Renal calcinosis and stone
PD30000245	M	0.8	2018	Eastern Area	Renal calcinosis and stone	*SLC3A1*	Renal calcinosis and stone
PD30000246	M	1.1	2018	Eastern Area	Renal calcinosis and stone	*SLC7A9*	Renal calcinosis and stone
PD11112870	M	6.0	2018	Eastern Area	GN	*COL4A5*	Alport syn.
PD11112239	F	11.0	2018	Eastern Area	SRNS	*ADCK4*	FSGS
PD11112238	M	5.0	2018	Eastern Area	SRNS	*CUBN*	FSGS
PD30001498	F	4.0	2018	Eastern Area	CAKUT	*1q21.1-21.2del*	CAKUT
PD11112229	F	2.2	2018	Eastern Area	GN	*COL4A3*	Alport syn.
PD11112887	F	5.8	2018	Eastern Area	Renal tubular disease	*SLC12A1*	TID
PD11112889	F	9.0	2018	Eastern Area	GN	*COL4A5*	Alport syn.
PD11112893	F	4.3	2018	Eastern Area	Renal tubular disease	*SLC12A3*	TID
PD11111803	M	12.0	2018	Eastern Area	SRNS	*CoQ2*	FSGS
PD11112898	M	10.0	2018	Eastern Area	CKDU	*NPHP1*	NPHP
PD11112899	M	2.0	2018	Eastern Area	GN	*COL4A5*	Alport syn.
PD11112904	M	2.8	2018	Eastern Area	GN	*CLCN5*	FSGS
PD11112907	M	0.6	2018	Eastern Area	GN	*CLCN5*	FSGS
PD11112908	M	5.6	2018	Eastern Area	SRNS	*ADCK4*	FSGS
PD11112911	F	3.3	2018	Eastern Area	SRNS	*WT1*	FSGS
PD11112913	M	2.3	2018	Eastern Area	GN	*COL4A5*	Alport syn.
PD11112915	M	3.8	2018	Eastern Area	GN	*COL4A5*	Alport syn.
PD11112916	F	1.7	2018	Eastern Area	CKDU	*NPHP3*	NPHP
PD11112917	M	3.5	2018	Eastern Area	GN	*CLCN5*	FSGS
PD11112920	M	11.0	2018	Eastern Area	GN	*MYH9*	FSGS
PD11112922	M	10.0	2018	Eastern Area	HUS	*CFHR1*	HUS
PD11112924	M	2.7	2018	Eastern Area	GN	*COL4A5*	Alport syn.
PD11112925	F	0.8	2018	Eastern Area	SRNS	*WT1*	FSGS
PD11112928	F	9.0	2018	Eastern Area	CKDU	*NPHP3*	NPHP
PD11112930	M	11.0	2018	Eastern Area	CKDU	*NPHP1*	NPHP
PD30001731	F	1.8	2018	Eastern Area	SRNS	*PLCE1*	FSGS
PD11112563	M	3.0	2018	Eastern Area	GN	*COL4A5*	Alport syn.
PD11112940	M	5.0	2018	Eastern Area	GN	*COL4A5*	Alport syn.
PD11112950	M	8.0	2018	Eastern Area	GN	*COL4A3*	Alport syn.
PD11112952	F	3.5	2018	Eastern Area	CKDU	*WDR19*	NPHP
PD11112954	M	5.8	2018	Eastern Area	Renal tubular disease	*ATP6V0A4*	TID
PD11112956	M	6.2	2018	Eastern Area	Renal tubular disease	*SLC12A3*	TID
PD11112957	M	9.9	2018	Eastern Area	GN	*COL4A5*	Alport syn.
PD11112959	M	5.9	2018	Eastern Area	GN	*COL4A5*	Alport syn.
PD11112961	M	7.3	2018	Eastern Area	GN	*COL4A5*	Alport syn.
PD11112963	F	8.8	2018	Eastern Area	Renal tubular disease	*SLC12A3*	TID
PD11112969	M	7.0	2018	Eastern Area	Renal tubular disease	*SLC12A3*	TID
PD11112973	F	7.0	2018	Eastern Area	cystic renal disease	*PKHD1*	cystic renal disease
PD11111877	M	14.0	2017	Eastern Area	CKDU	*NPHP1*	NPHP
PD30000236	M	10.0	2016	Western Area	Renal calcinosis and stone	*AGXT*	Renal calcinosis and stone
PD11111879	M	3.0	2018	Eastern Area	Renal tubular disease	*CLCN5*	TID
PD30000785	F	1.0	2017	Eastern Area	SRNS	*NPHS1*	FSGS
PD11111882	M	0.1	2017	Eastern Area	GN	*TTC21B*	FSGS
PD11111884	M	2.4	2017	Eastern Area	CKDU	*NPHP3*	NPHP
PD11111885	F	0.2	2018	Western Area	CKDU	*INVS*	NPHP
PD11111886	F	0.2	2017	Middle Region	Renal tubular disease	*SLC12A1*	TID
PD11111890	F	14.0	2018	Eastern Area	SRNS	*PAX2*	FSGS
PD30002063	F	9.0	2018	Eastern Area	SRNS	*ADCK4*	FSGS
PD11111895	F	11.0	2018	Eastern Area	GN	*COL4A3*	Alport syn.
PD11111899	F	16.0	2018	Eastern Area	CKDU	*NPHP1*	NPHP
PD11111901	M	15.0	2018	Eastern Area	GN	*COL4A5*	Alport syn.
PD11112662	F	14.0	2018	Eastern Area	CKDU	*RPGRIP1L*	NPHP
PD11111905	M	17.0	2018	Eastern Area	SRNS	*INF2*	FSGS
PD11111571	M	8.0	2018	Eastern Area	SRNS	*ADCK4*	FSGS
PD11111909	M	10.0	2018	Eastern Area	CKDU	*NPHP1*	NPHP
PD11111911	F	9.0	2018	Eastern Area	CKDU	*NPHP1*	NPHP
PD11111912	M	3.3	2018	Eastern Area	cystic renal disease	*PKHD1*	cystic renal disease
PD30000063	F	14.0	2017	Eastern Area	Renal tubular disease	*SLC4A1*	TID
PD30000067	M	0.8	2017	Eastern Area	Renal tubular disease	*SLC12A1*	TID
PD30000086	M	2.0	2016	Eastern Area	Renal tubular disease	*CLCN5*	TID
PD30000087	M	2.0	2016	Eastern Area	Renal tubular disease	*CLCN5*	TID
PD30000090	F	17.0	2017	Eastern Area	Renal tubular disease	*KLHL3*	TID
PD30000091	M	0.9	2018	Eastern Area	SRNS	*OCRL*	FSGS
PD30000092	M	2.1	2014	Eastern Area	SRNS	*OCRL*	FSGS
PD30000094	M	11.8	2017	Eastern Area	SRNS	*NUP107*	FSGS
PD30000095	F	5.6	2014	Eastern Area	SRNS	*ADCK4*	FSGS
PD30000160	M	0.7	2016	Eastern Area	cystic renal disease	*PKHD1*	cystic renal disease
PD30000097	M	3.8	2014	Eastern Area	GN	*COL4A5*	Alport syn.
PD30000099	M	9.0	2015	Eastern Area	GN	*COL4A5*	Alport syn.
PD30000100	M	10.5	2015	Eastern Area	GN	*COL4A3*	Alport syn.
PD30000101	F	11.9	2016	Eastern Area	HUS	*C3*	HUS
PD30000121	F	0.3	2018	Eastern Area	SRNS	*WT1*	FSGS
PD30000135	F	2.0	2018	Eastern Area	SRNS	*CD2AP*	FSGS
PD30000136	F	5.0	2018	Eastern Area	SRNS	*WT1*	FSGS
PD30000138	F	0.3	2018	Eastern Area	SRNS	*LAMB2*	FSGS
PD30000140	F	11.4	2018	Eastern Area	SRNS	*WT1*	FSGS
PD30000142	M	0.2	2018	Eastern Area	SRNS	*OCRL*	FSGS

PD30000145	F	3.1	2018	Eastern Area	GN	*COL4A5*	Alport syn.
PD30000146	M	6.0	2018	Eastern Area	GN	*MMACHC*	Metabolic disorders
PD30000147	M	4.8	2018	Eastern Area	SRNS	*MYH9*	FSGS
PD30000148	M	2.2	2018	Eastern Area	GN	*COL4A5*	Alport syn.
PD30000150	M	6.2	2016	Eastern Area	Renal tubular disease	*CLCN5*	TID
PD30000151	M	0.4	2016	Eastern Area	cystic renal disease	*BBS1*	cystic renal disease
PD30000152	F	1.9	2016	Eastern Area	GN	*COL4A5*	Alport syn.
PD30000153	M	12.0	2016	Eastern Area	GN	*COL4A5*	Alport syn.
PD30000154	F	4.5	2017	Eastern Area	GN	*COL4A5*	Alport syn.
PD30000156	M	6.0	2016	Eastern Area	Renal tubular disease	*SLC12A3*	TID
PD30000157	M	9.0	2018	Eastern Area	GN	*COL4A5*	Alport syn.
PD30000158	M	10.0	2018	Eastern Area	Renal tubular disease	*SLC12A3*	TID
PD30000159	F	5.0	2018	Eastern Area	Renal tubular disease	*SLC4A1*	TID
PD30000161	F	1.4	2017	Eastern Area	SRNS	*PLCE1*	FSGS
PD30000165	F	6.5	2017	Eastern Area	GN	*COL4A5*	Alport syn.
PD30000167	F	5.0	2017	Eastern Area	GN	*COL4A4*	Alport syn.
PD30000168	M	12.0	2017	Eastern Area	GN	*COL4A5*	Alport syn.
PD30000169	M	8.3	2015	Eastern Area	Renal tubular disease	*ATP6V1B1*	TID
PD30000170	M	7.5	2015	Eastern Area	Renal tubular disease	*SLC12A3*	TID
PD30000171	M	5.0	2015	Eastern Area	Renal tubular disease	*SLC4A1*	TID
PD11112970	M	2.6	2018	Eastern Area	SRNS	*PLCE1*	FSGS
PD11111534	F	15.1	2017	Eastern Area	Renal calcinosis and stone	*AGXT*	Renal calcinosis and stone
PD11111559	F	11.8	2017	Eastern Area	SRNS	*WT1*	FSGS
PD11111561	F	7.3	2017	Eastern Area	SRNS	*WT1*	FSGS
PD11111585	M	7.7	2017	Eastern Area	SRNS	*WT1*	FSGS
PD11111859	F	4.5	2018	Eastern Area	GN	*COL4A5*	Alport syn.
PD11111866	M	4.2	2018	Eastern Area	GN	*COL4A4*	Alport syn.
PD11112072	M	14.3	2018	Eastern Area	cystic renal disease	*PKD1*	cystic renal disease
PD11112112	M	10.6	2019	Eastern Area	Renal tubular disease	*SLC5A2*	TID
PD11112253	F	1.4	2018	Eastern Area	Renal calcinosis and stone	*AGXT*	Renal calcinosis and stone
PD11112687	M	16.0	2017	Eastern Area	SRNS	*NUP160*	FSGS
PD11112763	M	2.7	2019	Eastern Area	CAKUT	*Xq28*	CAKUT
PD111111321	M	13.2	2019	Eastern Area	CKDU	*CoQ2*	FSGS
PD11112974	M	10.3	2019	Eastern Area	GN	*COL4A4*	Alport syn.
PD11112976	M	4.9	2019	Eastern Area	GN	*NPHP3*	FSGS
PD11112977	M	1.5	2019	Eastern Area	SRNS	*NPHS1*	FSGS
PD11112978	M	2.8	2019	Eastern Area	CAKUT	*Xq22.2dul*	CAKUT
PD30002086	M	5.1	2019	Eastern Area	cystic renal disease	*PKD1*	cystic renal disease
PD11112990	F	1.5	2019	Eastern Area	Renal calcinosis and stone	*CYP24A1*	Renal calcinosis and stone
PD11112992	M	5.1	2019	Eastern Area	CAKUT	*NPHP1*	CAKUT
PD11112993	M	5.6	2019	Eastern Area	Renal tubular disease	*CLCN5*	TID
PD11112996	M	0.4	2019	Eastern Area	SRNS	*NPHS1*	FSGS
PD11112998	M	10.0	2019	Eastern Area	GN	*OCRL*	GN
PD11113011	M	0.5	2019	Eastern Area	CAKUT	*17q.12*	CAKUT
PD30000315	F	17.5	2019	Eastern Area	CKDU	*WDR19*	NPHP
PD30000316	F	2.4	2019	Eastern Area	CKDU	*LPIN1*	Metabolic disorders
PD30002037	M	3.6	2019	Eastern Area	SRNS	*NPHS1*	FSGS
PD30000348	M	12.5	2019	Eastern Area	GN	*COL4A4*	Alport syn.
PD30002038	M	4.7	2019	Eastern Area	CAKUT	*17p11.2*	CAKUT
PD111111401	F	5.0	2019	Eastern Area	SRNS	*SMARCAL1*	FSGS
PD30000356	F	3.0	2019	Eastern Area	cystic renal disease	*PKHD1*	cystic renal disease
PD30000588	F	7.8	2019	Eastern Area	CKDU	*TMEM67*	NPHP
PD30000591	F	6.8	2019	Eastern Area	GN	*COL4A3*	Alport syn.
PD30002036	F	1.8	2019	Eastern Area	SRNS	*WT1*	FSGS
PD30000597	M	13.8	2019	Eastern Area	SRNS	*MYH9*	FSGS
PD30000599	M	15.1	2019	Eastern Area	SRNS	*INF2*	FSGS
PD30000722	M	0.1	2019	Eastern Area	Renal tubular disease	*AVPR2*	TID
PD30002034	F	3.0	2019	Eastern Area	SRNS	*WT1*	FSGS
PD30000724	F	6.0	2019	Eastern Area	GN	*COL4A5*	Alport syn.
PD30000730	F	7.1	2019	Eastern Area	GN	*COL4A5*	Alport syn.
PD30000731	F	3.5	2019	Eastern Area	GN	*COL4A5*	Alport syn.
PD30000732	M	14.0	2019	Eastern Area	GN	*COL4A5*	Alport syn.
PD30002033	F	4.0	2019	Eastern Area	SRNS	*LAMB2*	FSGS
PD30000839	M	2.1	2019	Eastern Area	Renal tubular disease	*KCNJ1*	TID
PD30000842	F	2.0	2019	Eastern Area	Renal calcinosis and stone	*HOGA1*	Renal calcinosis and stone
PD30000843	M	3.5	2019	Eastern Area	Renal calcinosis and stone	*SLC34A1*	Renal calcinosis and stone
PD30000845	M	15.6	2019	Eastern Area	Renal calcinosis and stone	*KCNJ1*	Renal calcinosis and stone
PD30000846	M	1.8	2019	Eastern Area	Renal calcinosis and stone	*HOGA1*	Renal calcinosis and stone
PD30001016	M	6.1	2019	Eastern Area	CAKUT	*17q.12*	CAKUT
PD30001024	M	3.1	2019	Eastern Area	GN	*COL4A5*	Alport syn.
PD30001078	M	3.7	2019	Eastern Area	CAKUT	*ROBO2*	CAKUT
PD30001214	F	4.9	2019	Eastern Area	CKDU	*NPHP3*	NPHP
PD30001735	M	8.6	2020	Eastern Area	CKDU	*NPHP4*	NPHP
PD30001737	M	7.5	2020	Eastern Area	SRNS	*COL4A4*	FSGS
PD30001740	F	0.8	2020	Eastern Area	cystic renal disease	*NPHP3*	cystic renal disease
PD30001762	M	1.5	2020	Eastern Area	SRNS	*WT1*	FSGS
PD30001776	M	13.4	2020	Eastern Area	CAKUT	*Xq*	CAKUT
PD30001782	M	9.8	2020	Eastern Area	GN	*IFT172*	FSGS
PD30001783	M	7.4	2020	Eastern Area	Renal tubular disease	*SLC12A3*	TID
PD30001789	F	2.8	2020	Eastern Area	GN	*WDR19*	FSGS
PD30001793	M	1.5	2020	Eastern Area	cystic renal disease	*PKHD1*	cystic renal disease
PD30001798	M	8.2	2020	Eastern Area	GN	*COL4A5*	Alport syn.
PD30001799	F	8.2	2020	Eastern Area	GN	*COL4A3*	GN
PD30001800	F	7.6	2020	Eastern Area	GN	*COL4A5*	Alport syn.
PD30001801	F	15.7	2020	Eastern Area	GN	*COL4A3*	GN
PD30001938	F	7.2	2020	Eastern Area	CKDU	*ADCK4*	FSGS
PD30001943	M	11.9	2020	Eastern Area	SRNS	*NUP107*	FSGS
PD30001945	M	10.8	2020	Eastern Area	SRNS	*ADCK4*	FSGS
PD30001961	M	10.8	2020	Eastern Area	cystic renal disease	*PKD1*	cystic renal disease
PD30001964	M	11.6	2020	Eastern Area	CKDU	*COL4A5*	Alport syn.
PD30001967	M	11.6	2020	Eastern Area	CAKUT	*WT1*	CAKUT
PD30001999	M	5.6	2020	Eastern Area	SRNS	*PAX2*	FSGS
PD30002000	F	12.8	2020	Eastern Area	CKDU	*NPHP1*	NPHP
PD30002002	M	1.9	2020	Eastern Area	SRNS	*COQ6*	FSGS

PD30002031	F	12.2	2020	Eastern Area	SRNS	*WT1*	FSGS
PD30002044	M	11.7	2020	Eastern Area	SRNS	*PAX2*	FSGS
PD111111821	F	15.6	2020	Eastern Area	SRNS	*SMARCAL1*	FSGS
PD30002056	F	0.2	2020	Eastern Area	SRNS	*NPHS1*	FSGS
PD30002058	F	7.6	2020	Eastern Area	CKDU	*PAX2*	CAKUT
PD30002060	F	1.7	2020	Eastern Area	GN	*COL4A5*	GN
PD30002061	M	12.3	2020	Eastern Area	CKDU	*ADCK4*	FSGS
PD30002062	M	14.3	2020	Eastern Area	CKDU	*CEP290*	NPHP
PD30002097	F	15.8	2020	Eastern Area	CAKUT	*PAX2*	CAKUT
PD30002100	M	1.2	2020	Eastern Area	SRNS	*16p.13.11del*	FSGS
PD30002103	M	4.0	2020	Eastern Area	GN	*CLCN5*	FSGS
PD30002216	M	10.8	2020	Eastern Area	GN	*COL4A4*	Alport syn.
PD30002220	M	7.0	2020	Eastern Area	CKDU	*NPHP1*	NPHP
PD30002223	F	4.3	2020	Eastern Area	GN	*MMACHC*	Metabolic disorders
PD30000894	M	13.3	2019	Eastern Area	GN	*CLCN5*	FSGS
PD30000895	M	12.0	2019	Eastern Area	GN	*CLCN5*	FSGS
PD30000897	M	8.3	2019	Eastern Area	GN	*CLCN5*	FSGS
PD30001276	M	4.7	2019	Eastern Area	CKDU	*NPHP3*	NPHP
PD30001277	M	14.2	2019	Eastern Area	CKDU	*NPHP1*	NPHP
PD30001278	M	15.3	2019	Eastern Area	CKDU	*NPHP4*	NPHP
PD30001279	M	14.6	2019	Eastern Area	CKDU	*NPHP1*	NPHP
PD30001280	M	4.2	2019	Eastern Area	GN	*COL4A5*	Alport syn.
PD30001281	M	9.7	2019	Eastern Area	CKDU	*ADCK4*	FSGS
PD30001282	M	6.4	2019	Eastern Area	GN	*COL4A5*	Alport syn.
PD30001283	F	2.5	2019	Eastern Area	SRNS	*NPHS2*	FSGS
PD30000629	M	9.0	2019	Middle Region	GN	*COL4A4*	Alport syn.
PD30000630	F	12.0	2019	Middle Region	CAKUT	*PAX2*	CAKUT
PD30000631	F	6.1	2019	Middle Region	cystic renal disease	*PKD1*	cystic renal disease
PD30000633	M	5.1	2019	Middle Region	GN	*COL4A5*	Alport syn.
PD30000634	M	3.7	2019	Middle Region	GN	*COL4A5*	Alport syn.
PD30000641	F	8.3	2019	Middle Region	GN	*COL4A5*	Alport syn.
PD111111968	M	0.4	2020	Middle Region	CAKUT	*HNF1B*	CAKUT
PD30001867	F	12.5	2020	Middle Region	CAKUT	*PAX2*	CAKUT
PD30000916	M	2.8	2019	Eastern Area	Renal tubular disease	*CLCN5*	TID
PD30000787	M	7.4	2019	Middle Region	Renal tubular disease	*CLCN5*	TID
PD30000788	M	1.7	2019	Middle Region	SRNS	*COQ2*	FSGS
PD30000789	F	8.9	2019	Middle Region	GN	*COL4A5*	Alport syn.
PD30000790	F	11.6	2019	Middle Region	GN	*COL4A4*	Alport syn.
PD30000791	F	8.8	2019	Middle Region	GN	*COL4A5*	Alport syn.
PD30000793	F	5.9	2019	Middle Region	GN	*COL4A4*	Alport syn.
PD30000794	F	8.2	2019	Middle Region	GN	*COL4A4*	Alport syn.
PD30000795	M	10.6	2019	Middle Region	SRNS	*ADCK4*	FSGS
PD30000796	M	11.5	2019	Middle Region	GN	*COL4A5*	Alport syn.
PD30000797	F	11.8	2019	Middle Region	GN	*COL4A4*	Alport syn.
PD30000800	F	4.5	2019	Middle Region	SRNS	*TRPC6*	FSGS
PD30000802	M	5.4	2019	Middle Region	GN	*MMACHC*	Metabolic disorders
PD30000436	M	6.8	2019	Eastern Area	SRNS	*INF2*	FSGS
PD30000475	M	6.5	2019	Eastern Area	Renal tubular disease	*WNK1*	TID
PD30000480	M	10.3	2019	Eastern Area	GN	*COL4A4*	Alport syn.
PD30000481	M	8.6	2019	Eastern Area	SRNS	*ARHGAP24*	FSGS
PD30000484	M	16.5	2019	Eastern Area	SRNS	*NPHS1*	FSGS
PD30000491	M	8.1	2019	Eastern Area	SRNS	*COL4A3*	FSGS
PD30000539	F	6.6	2019	Eastern Area	SRNS	*KANK4*	FSGS
PD30000541	M	8.8	2019	Eastern Area	SRNS	*COL4A4*	FSGS
PD30000546	M	10.5	2019	Eastern Area	CAKUT	*ROBO2*	CAKUT
PD30000549	M	7.0	2019	Eastern Area	GN	*COL4A4*	Alport syn.
PD30000550	M	13.8	2019	Eastern Area	Renal tubular disease	*CLCN5*	TID
PD30000556	F	8.0	2019	Eastern Area	SRNS	*WT1*	FSGS
PD30000557	M	9.1	2019	Eastern Area	Renal tubular disease	*CLCN5*	TID
PD30000558	F	7.6	2019	Eastern Area	GN	*COL4A5*	Alport syn.
PD30000559	F	4.5	2019	Eastern Area	GN	*COL4A5*	Alport syn.
PD30000560	F	12.9	2019	Eastern Area	GN	*COL4A3*	Alport syn.
PD30000562	F	13.0	2019	Eastern Area	GN	*UMOD*	FSGS
PD30000563	M	10.0	2019	Eastern Area	GN	*COL4A5*	Alport syn.
PD30000565	M	10.6	2019	Eastern Area	SRNS	*WT1*	FSGS
PD30000566	M	13.0	2019	Eastern Area	GN	*COL4A4*	Alport syn.
PD30000569	M	15.6	2019	Eastern Area	GN	*COL4A5*	Alport syn.
PD30000570	M	15.3	2019	Eastern Area	Renal tubular disease	*AQP2*	TID
PD30000571	M	5.2	2019	Eastern Area	SRNS	*ANLN*	FSGS
PD30000575	M	14.5	2019	Eastern Area	GN	*GLA*	Metabolic disorders
PD30000577	F	7.0	2019	Eastern Area	GN	*COL4A5*	Alport syn.
PD30000580	F	3.9	2019	Eastern Area	Renal tubular disease	*SLC5A2*	TID
PD30000582	M	8.7	2019	Eastern Area	CAKUT	*PAX2*	CAKUT
PD30000583	M	10.0	2019	Eastern Area	CAKUT	*PAX2*	CAKUT
PD30000593	F	8.0	2019	Eastern Area	SRNS	*TRPC6*	FSGS
PD30000596	F	3.2	2019	Eastern Area	GN	*COL4A3*	Alport syn.
PD30001621	M	14.9	2020	Eastern Area	GN	*COL4A5*	Alport syn.
PD30001631	M	5.4	2020	Eastern Area	GN	*COL4A5*	Alport syn.
PD30001635	F	9.4	2020	Eastern Area	Renal calcinosis and stone	*AGXT*	Renal calcinosis and stone
PD30001637	F	3.6	2020	Eastern Area	SRNS	*WT1*	FSGS
PD30001651	M	11.6	2020	Eastern Area	SRNS	*DUOX2*	FSGS
PD30001653	F	3.7	2020	Eastern Area	GN	*COL4A5*	Alport syn.
PD30001656	F	3.8	2020	Eastern Area	GN	*COL4A5*	Alport syn.
PD30001657	M	14.4	2020	Eastern Area	GN	*COL4A5*	Alport syn.
PD30001664	M	4.9	2020	Eastern Area	GN	*COL4A5*	Alport syn.
PD30001668	M	2.6	2020	Eastern Area	SRNS	*WT1*	FSGS
PD30001677	F	13.6	2020	Eastern Area	SRNS	*PAX2*	FSGS
PD30001684	M	9.7	2020	Eastern Area	GN	*COL4A4*	Alport syn.
PD30001685	M	2.7	2020	Eastern Area	SRNS	*NPHS2*	FSGS
PD30001687	M	4.1	2020	Eastern Area	GN	*COL4A5*	Alport syn.
PD30001688	F	2.5	2020	Eastern Area	SRNS	*WT1*	FSGS
PD30001695	F	11.6	2020	Eastern Area	CAKUT	*GATA3*	CAKUT
PD30001696	F	4.1	2020	Eastern Area	GN	*COL4A5*	Alport syn.
PD30001700	M	6.9	2020	Eastern Area	SRNS	*COL4A3*	FSGS

PD30001703	M	14.3	2020	Eastern Area	HUS	*CFI*	HUS
PD30001705	F	6.8	2020	Eastern Area	GN	*CC2D2A*	FSGS
PD30001708	F	3.3	2020	Eastern Area	GN	*COL4A5*	Alport syn.
PD30001714	M	2.4	2020	Eastern Area	CKDU	*HNF1B*	CAKUT
PD30001264	M	10.0	2019	Middle Region	GN	*COL4A4*	Alport syn.
PD30001265	F	12.9	2019	Middle Region	GN	*WDR19*	FSGS
PD30001266	F	6.6	2019	Middle Region	GN	*COL4A4*	Alport syn.
PD111112146	M	15.3	2019	Middle Region	CKDU	*NPHP1*	NPHP
PD30001268	M	6.0	2019	Middle Region	GN	*COL4A4*	Alport syn.
PD30001269	F	5.0	2019	Middle Region	GN	*COL4A5*	Alport syn.
PD30001270	M	10.9	2019	Middle Region	GN	*COL4A5*	Alport syn.
PD30001272	F	12.3	2019	Middle Region	GN	*COL4A4*	Alport syn.
PD30001273	M	2.6	2019	Middle Region	GN	*COL4A3*	Alport syn.
PD30001275	M	9.6	2019	Middle Region	GN	*COL4A4*	Alport syn.
PD30002071	M	6.0	2020	Middle Region	CKDU	*PAX2*	CAKUT
PD30002075	M	1.8	2020	Middle Region	SRNS	*INF2*	FSGS
PD30002076	M	7.3	2020	Middle Region	GN	*COL4A5*	Alport syn.
PD30002079	M	2.9	2020	Middle Region	GN	*OCRL*	GN
PD30002080	F	1.7	2020	Middle Region	GN	*COL4A4*	GN
PD30002081	F	10.3	2020	Middle Region	GN	*COL4A3*	Alport syn.
PD30002083	M	3.5	2020	Middle Region	GN	*COL4A5*	Alport syn.
PD30002084	M	11.9	2020	Middle Region	GN	*COL4A5*	Alport syn.
PD30000267	M	9.6	2019	Eastern Area	GN	*COL4A5*	Alport syn.
PD30000268	F	7.5	2019	Eastern Area	GN	*COL4A4*	GN
PD30000275	F	8.9	2019	Eastern Area	GN	*COL4A3*	Alport syn.
PD30000277	M	11.0	2019	Eastern Area	SRNS	*COL4A4*	FSGS
PD30000286	F	14.3	2019	Eastern Area	SRNS	*WT1*	FSGS
PD30000292	M	12.7	2019	Eastern Area	GN	*COL4A5*	Alport syn.
PD30000293	F	9.6	2019	Eastern Area	GN	*COL4A3*	GN
PD30000294	M	7.7	2019	Eastern Area	Renal tubular disease	*CLCN5*	TID
PD30000295	F	9.5	2019	Eastern Area	GN	*COL4A5*	Alport syn.
PD30000298	F	7.6	2019	Eastern Area	SRNS	*WT1*	FSGS
PD30000325	M	4.5	2019	Eastern Area	GN	*COL4A5*	Alport syn.
PD30000326	M	2.7	2019	Eastern Area	GN	*COL4A5*	Alport syn.
PD30000333	M	7.4	2019	Eastern Area	HUS	*MMACHC*	Metabolic disorders
PD30000418	M	9.2	2019	Eastern Area	SRNS	*SMARCAL1*	FSGS
PD30000422	M	8.0	2019	Eastern Area	GN	*COL4A5*	Alport syn.
PD30000424	M	11.0	2019	Eastern Area	GN	*COL4A5*	Alport syn.
PD30000426	F	8.4	2019	Eastern Area	GN	*COL4A5*	Alport syn.
PD30000439	F	0.8	2019	Eastern Area	SRNS	*NPHS1*	FSGS
PD30000444	M	3.1	2019	Eastern Area	GN	*COL4A5*	Alport syn.
PD30000445	M	3,2	2019	Eastern Area	GN	*COL4A5*	Alport syn.
PD30000446	F	4.3	2019	Eastern Area	GN	*COL4A5*	Alport syn.
PD30000449	M	3.6	2019	Eastern Area	SRNS	*ADCK4*	FSGS
PD30000453	M	9.4	2019	Eastern Area	GN	*COL4A5*	Alport syn.
PD30000457	M	11.7	2019	Eastern Area	GN	*COL4A3*	Alport syn.
PD30000459	F	7.7	2019	Eastern Area	GN	*COL4A5*	Alport syn.
PD30000464	M	5.4	2019	Eastern Area	GN	*COL4A5*	Alport syn.
PD30000466	M	8.2	2019	Eastern Area	Renal tubular disease	*CLCN5*	TID
PD30000467	M	5.1	2019	Eastern Area	GN	*COL4A5*	Alport syn.
PD30000675	M	1.2	2019	Eastern Area	SRNS	*CoQ2*	FSGS
PD30000680	M	10.7	2019	Eastern Area	Renal tubular disease	*CLCN5*	FSGS
PD30001934	F	4.6	2020	Eastern Area	GN	*COL4A5*	Alport syn.
PD30001953	F	11.6	2020	Eastern Area	GN	*COL4A5*	Alport syn.
PD30001954	M	13.3	2020	Eastern Area	GN	*MMACHC*	Metabolic disorders
PD30000750	M	10.0	2019	North-Eastern Ar	cystic renal disease	*PKHD1*	cystic renal disease
PD30000752	M	13.0	2019	North-Eastern Ar	GN	*MMACHC*	Metabolic disorders
PD30000753	F	13.5	2019	North-Eastern Ar	ESRD	*MMACHC*	Metabolic disorders
PD30000754	M	14.7	2019	North-Eastern Ar	GN	*COL4A4*	Alport syn.
PD30000756	M	11.2	2019	North-Eastern Ar	GN	*COL4A5*	Alport syn.
PD30000757	M	17.1	2019	North-Eastern Ar	GN	*COL4A3*	GN
PD30000758	M	5.5	2019	North-Eastern Ar	GN	*COL4A5*	Alport syn.
PD30000759	F	5.9	2019	North-Eastern Ar	GN	*COL4A3*	GN
PD30000760	F	6.4	2019	North-Eastern Ar	GN	*COL4A4*	GN
PD30000761	F	5.4	2019	North-Eastern Ar	SRNS	*CD2AP*	FSGS
PD30000762	F	18.9	2019	North-Eastern Ar	SRNS	*COL4A4*	FSGS
PD30000763	F	12.3	2019	North-Eastern Ar	GN	*COL4A3*	GN
PD30000764	M	10.3	2019	North-Eastern Ar	GN	*COL4A4*	GN
PD30000765	M	3.6	2019	North-Eastern Ar	GN	*COL4A5*	Alport syn.
PD30000766	F	2.6	2019	North-Eastern Ar	SRNS	*NPHS1*	FSGS
PD30000767	M	5.8	2019	North-Eastern Ar	GN	*COL4A4*	GN
PD30000768	M	4.3	2019	North-Eastern Ar	GN	*COL4A3*	GN
PD30000770	F	5.8	2019	North-Eastern Ar	GN	*COL4A4*	GN
PD30000771	M	11.3	2019	North-Eastern Ar	GN	*COL4A3*	GN
PD30000772	M	15.7	2019	North-Eastern Ar	Renal tubular disease	*SLC12A3*	TID
PD30000773	M	1.0	2019	North-Eastern Ar	GN	*LAMB2*	FSGS
PD30000774	F	0.5	2019	North-Eastern Ar	SRNS	*WT1*	FSGS
PD30000775	F	2.8	2019	North-Eastern Ar	cystic renal disease	*PKHD1*	cystic renal disease
PD30000776	F	11.2	2019	North-Eastern Ar	GN	*COL4A4*	Alport syn.
PD30000777	M	3.0	2019	North-Eastern Ar	SRNS	*ADCK4*	FSGS
PD30000007	M	6.7	2018	Eastern Area	GN	*COL4A5*	Alport syn.
PD30002065	M	2.6	2020	Eastern Area	GN	*COL4A5*	Alport syn.
PD30002066	M	11.4	2020	Eastern Area	CKDU	*PAX2*	CAKUT
PD30002068	F	13.9	2020	Eastern Area	GN	*COL4A3*	FSGS
PD30001223	F	8.7	2019	Western Area	Renal tubular disease	*CLCNKB*	TID
PD30001252	M	15.5	2019	Western Area	Renal tubular disease	*SLC12A3*	TID
PD30001254	M	13.4	2019	Western Area	Renal tubular disease	*SLC12A3*	TID
PD30001255	M	8.3	2019	Western Area	Renal tubular disease	*CLCNKB*	TID
PD30001256	F	19.9	2019	Western Area	GN	*COL4A5*	Alport syn.
PD30001257	M	14.9	2019	Western Area	GN	*COL4A5*	Alport syn.
PD30001258	M	8.8	2019	Western Area	GN	*COL4A5*	Alport syn.
PD30001259	M	5.3	2019	Western Area	GN	*COL4A5*	Alport syn.
PD30001260	M	7.5	2019	Western Area	GN	*COL4A5*	Alport syn.
PD30001261	F	11.0	2019	Western Area	GN	*COL4A5*	Alport syn.

PD30001262	F	8.9	2019	Western Area	GN	*COL4A5*	Alport syn.
PD30001287	F	12.0	2020	Western Area	GN	*COL4A5*	Alport syn.
PD30001288	F	11.7	2020	Western Area	GN	*COL4A5*	Alport syn.
PD30001573	M	5.0	2020	Western Area	GN	*COL4A5*	Alport syn.
PD30001574	F	9.3	2020	Western Area	GN	*COL4A5*	Alport syn.
PD30001575	M	13.2	2020	Western Area	GN	*COL4A5*	Alport syn.
PD30001576	M	5.3	2020	Western Area	GN	*COL4A4*	Alport syn.
PD30001577	M	4.9	2020	Western Area	GN	*COL4A5*	Alport syn.
PD30001578	M	8.2	2020	Western Area	GN	*COL4A5*	Alport syn.
PD30001579	F	5.4	2020	Western Area	GN	*COL4A3*	Alport syn.
PD30001580	F	7.3	2020	Western Area	GN	*COL4A3*	Alport syn.
PD30001581	M	15.1	2020	Western Area	GN	*COL4A3*	Alport syn.
PD30001582	F	15.9	2020	Western Area	GN	*COL4A3*	Alport syn.
PD30001583	M	5.3	2020	Western Area	GN	*COL4A3*	Alport syn.
PD30001585	F	5.9	2020	Western Area	GN	*COL4A4*	Alport syn.
PD30001586	F	7.1	2020	Western Area	GN	*COL4A4*	Alport syn.
PD30001587	F	11.1	2020	Western Area	GN	*COL4A4*	Alport syn.
PD30001589	M	5.6	2020	Western Area	GN	*COL4A5*	Alport syn.
PD30001591	M	6.4	2020	Western Area	GN	*COL4A5*	Alport syn.
PD30001593	M	13.0	2020	Western Area	GN	*COL4A5*	Alport syn.
PD30000864	M	2.8	2019	Eastern Area	GN	*COL4A3*	Alport syn.
PD30000865	M	9.4	2019	Eastern Area	GN	*COL4A3*	Alport syn.
PD30000867	F	9.1	2019	Eastern Area	GN	*COL4A3*	Alport syn.
PD30000872	M	12.5	2019	Eastern Area	GN	*COL4A4*	Alport syn.
PD30000873	F	7.2	2019	Eastern Area	GN	*COL4A4*	Alport syn.
PD30000879	F	12.0	2019	Eastern Area	GN	*COL4A5*	Alport syn.
PD30000925	M	9.4	2019	Eastern Area	GN	*COL4A5*	Alport syn.
PD30000928	F	14.7	2019	Eastern Area	GN	*COL4A5*	Alport syn.
PD30000929	M	14.8	2019	Eastern Area	GN	*COL4A5*	Alport syn.
PD30000930	F	7.0	2019	Eastern Area	GN	*COL4A5*	Alport syn.
PD30000931	M	7.0	2019	Eastern Area	GN	*COL4A5*	Alport syn.
PD30000932	F	9.8	2019	Eastern Area	GN	*COL4A5*	Alport syn.
PD30000933	M	5.7	2019	Eastern Area	GN	*COL4A5*	Alport syn.
PD30000934	F	5.8	2019	Eastern Area	GN	*COL4A5*	Alport syn.
PD30000935	F	5.8	2019	Eastern Area	GN	*COL4A5*	Alport syn.
PD30000936	M	5.2	2019	Eastern Area	GN	*COL4A5*	Alport syn.
PD30000937	M	5.1	2019	Eastern Area	GN	*COL4A5*	Alport syn.
PD30000938	M	3.8	2019	Eastern Area	GN	*COL4A5*	Alport syn.
PD30000940	F	17.2	2019	Eastern Area	GN	*COL4A5*	Alport syn.
PD30000941	F	8.0	2019	Eastern Area	GN	*COL4A5*	Alport syn.
PD30000942	M	11.3	2019	Eastern Area	GN	*COL4A5*	Alport syn.
PD30000943	M	3.3	2019	Eastern Area	GN	*COL4A5*	Alport syn.
PD30000944	M	15.6	2019	Eastern Area	GN	*COL4A5*	Alport syn.
PD30000945	F	8.6	2019	Eastern Area	GN	*COL4A5*	Alport syn.
PD30001226	M	17.0	2019	Eastern Area	GN	*COL4A5*	Alport syn.
PD30001230	F	8.0	2019	Eastern Area	cystic renal disease	*PKD1*	cystic renal disease
PD30001232	F	9.1	2019	Eastern Area	cystic renal disease	*PKD1*	cystic renal disease
PD30001240	F	6.1	2019	Eastern Area	cystic renal disease	*PKHD1*	cystic renal disease
PD30001241	F	7.5	2019	Eastern Area	cystic renal disease	*PKHD1*	cystic renal disease
PD30001242	M	10.3	2019	Eastern Area	cystic renal disease	*PKD1*	cystic renal disease
PD30001244	F	10.8	2019	Eastern Area	cystic renal disease	*PKHD1*	cystic renal disease
PD30001245	M	11.8	2019	Eastern Area	cystic renal disease	*PKD1*	cystic renal disease
PD30001246	F	12.6	2019	Eastern Area	cystic renal disease	*PKD1*	cystic renal disease
PD30001248	M	13.2	2019	Eastern Area	cystic renal disease	*PKD1*	cystic renal disease
PD30001249	M	13.5	2019	Eastern Area	cystic renal disease	*PKD1*	cystic renal disease
PD30001567	F	13.5	2020	Eastern Area	GN	*COL4A5*	Alport syn.
PD30001569	M	12.3	2020	Eastern Area	GN	*COL4A5*	Alport syn.
PD30000082	F	6.6	2019	Eastern Area	SRNS	*WT1*	FSGS
PD30000084	M	4.7	2019	Eastern Area	Renal tubular disease	*CLCNKB*	TID
PD30000088	F	3.0	2019	Eastern Area	Renal tubular disease	*SLC12A1*	TID
PD30000089	M	3.5	2019	Eastern Area	Renal tubular disease	*SLC12A1*	TID
PD30000093	M	5.0	2019	Eastern Area	GN	*OCRL*	GN
PD30000046	M	4.3	2019	Middle Region	cystic renal disease	*PKD1*	cystic renal disease
PD30000053	F	3.4	2019	Middle Region	GN	*COL4A4*	Alport syn.
PD30000054	F	4.8	2019	Middle Region	HUS	*C3*	HUS
PD30000057	F	2.4	2019	Middle Region	GN	*COL4A5*	Alport syn.
PD30000493	M	8.9	2019	Eastern Area	Renal tubular disease	*SLC12A3*	TID
PD30000494	F	1.9	2019	Eastern Area	cystic renal disease	*PKD1*	cystic renal disease
PD30000496	M	15.0	2019	Eastern Area	cystic renal disease	*PKD1*	cystic renal disease
PD30000497	F	11.2	2019	Eastern Area	GN	*COL4A4*	Alport syn.
PD30000498	M	3.9	2019	Eastern Area	cystic renal disease	*PKHD1*	cystic renal disease
PD30000499	F	9.8	2019	Eastern Area	GN	*COL4A4*	Alport syn.
PD30000500	F	6.4	2019	Eastern Area	GN	*COL4A4*	Alport syn.
PD30000501	M	12.6	2019	Eastern Area	cystic renal disease	*PKD1*	cystic renal disease
PD30000502	M	9.6	2019	Eastern Area	GN	*CLCN5*	FSGS
PD30000503	F	1.6	2019	Eastern Area	Renal calcinosis and stone	*HOGA1*	Renal calcinosis and stone
PD30000504	M	5.0	2019	Eastern Area	Renal tubular disease	*CLCN5*	TID
PD30000505	F	7.3	2019	Eastern Area	GN	*COL4A4*	Alport syn.
PD30000506	M	7.2	2019	Eastern Area	GN	*COL4A4*	Alport syn.
PD30000507	M	6.4	2019	Eastern Area	GN	*COL4A5*	Alport syn.
PD30000509	M	13.3	2019	Eastern Area	Renal tubular disease	*SLC12A3*	TID
PD30000513	M	8.5	2019	Eastern Area	GN	*COL4A5*	Alport syn.
PD30000515	F	4.6	2019	Eastern Area	GN	*COL4A5*	Alport syn.
PD30000516	M	11.6	2019	Eastern Area	CAKUT	*TBX18*	CAKUT
PD30000517	M	6.7	2019	Eastern Area	cystic renal disease	*PKD2*	cystic renal disease
PD30000519	F	10.6	2019	Eastern Area	GN	*COL4A4*	Alport syn.
PD30001814	F	13.4	2020	Eastern Area	CAKUT	*NPHP1*	CAKUT
PD30001822	M	2.8	2020	Eastern Area	CAKUT	*PAX2*	CAKUT
PD30001826	M	7.8	2020	Eastern Area	cystic renal disease	*PKD1*	cystic renal disease
PD30001827	M	3.1	2020	Eastern Area	CAKUT	*Yqh+,inv(5)(p15.1q11.2)*	CAKUT
PD30001829	F	1.0	2020	Eastern Area	CAKUT	*17q12*	CAKUT
PD30001833	F	3.4	2020	Eastern Area	CKDU	*NPHP1*	NPHP
PD30001834	M	6.7	2020	Eastern Area	cystic renal disease	*PKD2*	cystic renal disease
PD30001835	F	6.3	2020	Eastern Area	cystic renal disease	*PKD1*	cystic renal disease

PD30001836	M	1.8	2020	Eastern Area	CAKUT	*PKHD1*	CAKUT
PD30001839	M	9.8	2020	Eastern Area	cystic renal disease	*PKD1*	cystic renal disease
PD30001844	M	5.6	2020	Eastern Area	cystic renal disease	*PKD1*	cystic renal disease
PD30001986	F	8.4	2020	Eastern Area	Renal tubular disease	*SLC12A3*	TID
PD30001993	F	9.9	2020	Eastern Area	Renal tubular disease	*SLC12A3*	TID
PD30001994	F	17.1	2020	Eastern Area	Renal tubular disease	*SLC12A3*	TID
PD30001995	M	12.6	2020	Eastern Area	Renal tubular disease	*SLC12A3*	TID
PD30001996	M	12.8	2020	Eastern Area	Renal tubular disease	*SLC12A3*	TID
PD30001997	M	12.8	2020	Eastern Area	Renal tubular disease	*SLC12A3*	TID
PD30001998	M	15.5	2020	Eastern Area	Renal tubular disease	*SLC12A3*	TID
PD30002007	F	4.2	2020	Eastern Area	Renal tubular disease	*SLC12A3*	TID
PD30002009	F	2.9	2020	Eastern Area	Renal tubular disease	*SLC12A3*	TID
PD30002010	M	8.1	2020	Eastern Area	Renal tubular disease	*SLC12A3*	TID
PD30002011	F	11.0	2020	Eastern Area	Renal tubular disease	*SLC12A3*	TID
PD30000367	M	10.1	2019	Eastern Area	GN	*COL4A5*	Alport syn.
PD30000368	M	9.2	2019	Eastern Area	GN	*COL4A5*	Alport syn.
PD30000369	M	14.4	2019	Eastern Area	GN	*COL4A5*	Alport syn.
PD30000370	M	8.4	2019	Eastern Area	GN	*COL4A5*	Alport syn.
PD30000371	M	10.4	2019	Eastern Area	GN	*COL4A5*	Alport syn.
PD30000372	M	6.9	2019	Eastern Area	GN	*COL4A5*	Alport syn.
PD30000373	M	7.2	2019	Eastern Area	GN	*COL4A5*	Alport syn.
PD30000374	F	10.7	2019	Eastern Area	GN	*COL4A5*	Alport syn.
PD30000375	F	0.7	2019	Eastern Area	GN	*COL4A5*	Alport syn.
PD30000376	M	6.1	2019	Eastern Area	GN	*COL4A5*	Alport syn.
PD30000377	F	6.3	2019	Eastern Area	GN	*COL4A5*	Alport syn.
PD30000378	F	5.0	2019	Eastern Area	GN	*COL4A5*	Alport syn.
PD30000379	M	9.2	2019	Eastern Area	GN	*COL4A4*	Alport syn.
PD30000380	F	11.6	2019	Eastern Area	GN	*COL4A4*	Alport syn.
PD30000381	F	5.6	2019	Eastern Area	GN	*COL4A3*	Alport syn.
PD30000382	F	15.7	2019	Eastern Area	GN	*COL4A3*	Alport syn.
PD30000383	F	7.6	2019	Eastern Area	GN	*COL4A3*	Alport syn.
PD30000384	M	5.0	2019	Eastern Area	SRNS	*NPHS1*	FSGS
PD30000386	M	8.2	2019	Eastern Area	SRNS	*NPHS1*	FSGS
PD30001870	M	6.1	2020	Eastern Area	GN	*COL4A5*	Alport syn.
PD30001871	F	6.6	2020	Eastern Area	GN	*COL4A5*	Alport syn.
PD30001872	F	6.6	2020	Eastern Area	GN	*COL4A3*	Alport syn.
PD30001873	F	7.0	2020	Eastern Area	GN	*COL4A5*	Alport syn.
PD30001874	F	7.0	2020	Eastern Area	GN	*COL4A4*	Alport syn.
PD30001875	F	17.0	2020	Eastern Area	GN	*COL4A5*	Alport syn.
PD30001876	F	11.2	2020	Eastern Area	GN	*COL4A5*	Alport syn.
PD30001877	F	11.3	2020	Eastern Area	GN	*COL4A5*	Alport syn.
PD30001878	M	10.1	2020	Eastern Area	GN	*COL4A5*	Alport syn.
PD30001879	M	8.6	2020	Eastern Area	GN	*COL4A5*	Alport syn.
PD30001880	M	9.8	2020	Eastern Area	GN	*COL4A5*	Alport syn.
PD30001881	M	13.1	2020	Eastern Area	GN	*COL4A5*	Alport syn.
PD30001882	F	13.6	2020	Eastern Area	GN	*COL4A4*	Alport syn.
PD30001883	M	10.0	2020	Eastern Area	GN	*COL4A5*	Alport syn.
PD30001884	M	11.0	2020	Eastern Area	GN	*COL4A5*	Alport syn.
PD30001885	M	8.0	2020	Eastern Area	GN	*COL4A5*	Alport syn.
PD30001886	M	13.7	2020	Eastern Area	GN	*COL4A5*	Alport syn.
PD30001888	F	9.5	2020	Eastern Area	GN	*COL4A5*	Alport syn.
PD30001889	F	6.9	2020	Eastern Area	GN	*COL4A3*	Alport syn.
PD30001890	F	15.4	2020	Eastern Area	GN	*COL4A5*	Alport syn.
PD30001891	M	8.0	2020	Eastern Area	GN	*COL4A5*	Alport syn.
PD30001892	M	12.3	2020	Eastern Area	GN	*COL4A5*	Alport syn.
PD30001893	F	7.0	2020	Eastern Area	GN	*COL4A5*	Alport syn.
PD30001894	M	7.4	2020	Eastern Area	GN	*COL4A3*	Alport syn.
PD30001895	M	11.0	2020	Eastern Area	GN	*COL4A4*	Alport syn.
PD30001896	M	6.8	2020	Eastern Area	GN	*COL4A4*	Alport syn.
PD30001897	M	11.8	2020	Eastern Area	GN	*COL4A3*	Alport syn.
PD30001898	M	7.2	2020	Eastern Area	GN	*COL4A5*	Alport syn.
PD30001900	M	17.0	2020	Eastern Area	GN	*COL4A4*	Alport syn.
PD30001901	M	6.3	2020	Eastern Area	GN	*COL4A5*	Alport syn.
PD30001902	F	9.8	2020	Eastern Area	GN	*COL4A4*	Alport syn.
PD30001903	M	12.4	2020	Eastern Area	GN	*COL4A5*	Alport syn.
PD30001904	F	8.4	2020	Eastern Area	GN	*COL4A5*	Alport syn.
PD30001905	M	6.9	2020	Eastern Area	GN	*COL4A5*	Alport syn.
PD30001906	F	7.5	2020	Eastern Area	GN	*COL4A5*	Alport syn.
PD30001908	M	4.7	2020	Eastern Area	GN	*COL4A5*	Alport syn.
PD30000408	M	6.5	2019	Eastern Area	GN	*COL4A5*	Alport syn.
PD30000411	F	4.9	2019	Eastern Area	SRNS	*INF2*	FSGS
PD30000412	M	8.0	2019	Eastern Area	GN	*COL4A5*	Alport syn.
PD30000413	M	7.2	2019	Eastern Area	GN	*COL4A5*	Alport syn.
PD30000470	F	4.7	2019	Eastern Area	Renal calcinosis and stone	*HOGA1*	Renal calcinosis and stone
PD30000472	M	9.3	2019	Eastern Area	GN	*COL4A4*	Alport syn.
PD30000427	F	11.4	2019	Eastern Area	GN	*COL4A5*	Alport syn.
PD30000433	M	11.0	2019	Eastern Area	Renal tubular disease	*SLC12A3*	TID
PD30000606	M	2.3	2019	Eastern Area	Renal tubular disease	*CLCN5*	TID
PD30000609	M	9.5	2019	Eastern Area	GN	*OCRL*	GN
PD30000610	F	9.2	2019	Eastern Area	Renal tubular disease	*SLC12A1*	TID
PD30000611	M	3.8	2019	Eastern Area	Renal tubular disease	*OCRL*	TID
PD30000612	F	9.2	2019	Eastern Area	SRNS	*ACTN4*	FSGS
PD30000613	F	7.0	2019	Eastern Area	GN	*COL4A5*	Alport syn.
PD30000614	M	10.9	2019	Eastern Area	GN	*OCRL*	GN
PD30000657	F	10.0	2019	Eastern Area	CAKUT	*PAX2*	CAKUT
PD30000661	M	9.4	2019	Eastern Area	Renal tubular disease	*CLCN5*	FSGS
PD30000662	M	12.9	2019	Eastern Area	GN	*COL4A5*	Alport syn.
PD30000663	F	2.6	2019	Eastern Area	GN	*COL4A5*	Alport syn.
PD30001919	F	5.5	2020	Eastern Area	GN	*COL4A5*	Alport syn.
PD30001921	F	2.6	2020	Eastern Area	GN	*COL4A5*	Alport syn.
PD30000700	M	3.8	2019	North-Eastern Ar	GN	*COL4A5*	Alport syn.
PD30000912	M	1.9	2019	North-Eastern Ar	Renal calcinosis and stone	*AGXT*	Renal calcinosis and stone
PD30000684	F	9.1	2019	Eastern Area	ESRD	*MMACHC*	Metabolic disorders
PD30000685	M	14.9	2019	Eastern Area	GN	*COL4A5*	Alport syn.

PD30000686	F	14.7	2019	Eastern Area	GN	*COL4A5*	Alport syn.
PD30000687	M	6.8	2019	Eastern Area	GN	*COL4A5*	Alport syn.
PD30000688	F	11.6	2019	Eastern Area	GN	*COL4A4*	Alport syn.
PD30000689	M	13.4	2019	Eastern Area	GN	*COL4A4*	Alport syn.
PD30000690	F	12.6	2019	Eastern Area	GN	*COL4A5*	Alport syn.
PD30000691	M	13.6	2019	Eastern Area	GN	*COL4A5*	Alport syn.
PD30000808	F	8.6	2019	Eastern Area	SRNS	*11p.13del*	FSGS
PD30000810	M	7.2	2019	Eastern Area	GN	*COL4A4*	Alport syn.
PD30000812	M	8.7	2019	Eastern Area	GN	*COL4A4*	GN
PD30000814	M	6.4	2019	Eastern Area	GN	*COL4A5*	Alport syn.
PD30000815	M	14.5	2019	Eastern Area	CAKUT	*PAX2*	CAKUT
PD30000817	M	7.4	2019	Eastern Area	GN	*COL4A3*	Alport syn.
PD30000820	F	3.7	2019	Eastern Area	GN	*COL4A4*	Alport syn.
PD30000821	F	2.3	2019	Eastern Area	Renal tubular disease	*PHEX*	TID
PD30000856	M	3.1	2019	Eastern Area	SRNS	*ACTN4*	FSGS
PD30000859	F	6.1	2019	Eastern Area	SRNS	*INF2*	FSGS
PD30000861	M	6.1	2019	Eastern Area	GN	*COL4A5*	Alport syn.
PD30000862	F	6.2	2019	Eastern Area	cystic renal disease	*PKD1*	cystic renal disease
PD30000863	F	3.0	2019	Eastern Area	SRNS	*NPHS1*	FSGS
PD30000906	M	8.9	2019	Middle Region	SRNS	*ADCK4*	FSGS
PD30000959	F	0.8	2019	Middle Region	GN	*COL4A5*	Alport syn.
PD30000960	M	0.2	2019	Middle Region	GN	*COL4A5*	Alport syn.
PD30000962	M	0.5	2019	Middle Region	Renal tubular disease	*SLC12A1*	TID
PD30000963	F	0.5	2019	Middle Region	SRNS	*WT1*	FSGS
PD30000965	M	5,4	2019	Middle Region	Renal tubular disease	*SLC12A3*	TID
PD30000907	M	3.7	2019	Eastern Area	CAKUT	*KCNJ1*	CAKUT
PD30000913	M	7.4	2019	Western Area	Renal tubular disease	*CLCN5*	TID
PD30001284	F	7.9	2019	Eastern Area	CKDU	*NPHP12*	NPHP
PD30001285	M	7.2	2019	Eastern Area	CKDU	*NPHP3*	NPHP
PD30001566	F	11.2	2020	Eastern Area	SRNS	*NPHS1*	FSGS
PD30001603	F	5.2	2020	Eastern Area	GN	*COL4A3*	Alport syn.
PD30001604	M	5.8	2020	Eastern Area	GN	*COL4A5*	Alport syn.
PD30001605	M	10.5	2020	Eastern Area	GN	*COL4A4*	Alport syn.
PD30001690	F	10.5	2020	Eastern Area	GN	*COL4A3*	Alport syn.
PD30002122	F	1.1	2020	Eastern Area	cystic renal disease	*PKHD1*	cystic renal disease
PD30002231	M	3.0	2020	Eastern Area	SRNS	*COL4A4*	FSGS
PD30002249	M	4.0	2020	Eastern Area	Renal tubular disease	*CLCN5*	FSGS
PD30002262	F	5.0	2020	Eastern Area	GN	*COL4A3*	GN
PD30002269	F	4.0	2020	Eastern Area	SRNS	*WT1*	FSGS
PD30002270	F	1.0	2020	Eastern Area	GN	*COL4A5*	Alport syn.
PD30002285	M	4.0	2020	Eastern Area	GN	*COL4A5*	Alport syn.
PD30002286	M	8.0	2020	Eastern Area	GN	*CLCN5*	FSGS
PD30002296	M	6.0	2020	Eastern Area	GN	*GLA*	Metabolic disorders
PD30002297	F	3.0	2020	Eastern Area	GN	*GLA*	Metabolic disorders
PD30002301	M	2.0	2020	Eastern Area	GN	*CLCN5*	FSGS

All the details of phenotype and pathogenic varinants you can index the ID number by log in the Chinese Children Genetic Kidney Disease Database (CCGKDD, www. ccgkdd.com.cn).
Alport syn., Alport syndrome; CAKUT, congenital anomalies of the kidney and urinary tract; CKDu, CKD of unknown etiology; FSGS, focal segmental glomerular sclerosis; GN; glomerulonephritis; NPHP, nephronophthisis; SRNS, steroid resistant nephrotic syndrome; TID, tubulointerstitial disease.

Online resource 5. Percentage of complete data with its breakdown and incomplete data

Data categories	Complete data					Incomplete data
	Legible data				Illegible data	
	Incorrectly coded data	Inappropriate data	Unrecognized data	Valid data		
Site number				100.0		0.0
Creation time				100.0		0.0
ID				100.0		0.0
register unit				100.0		0.0
Physician				100.0		0.0
gender				100.0		0.0
date of birth				100.0		0.0
age onset		0.1		99.2		0.7
Progression to ESRD age				99.4		0.6
Family history	2.8			97.0		0.2
Clinical diagnosis	5.4			93.7		0.9
Diagnostic classification	3.6			95.5		0.9
Note for diagnosis		0.2		99.7		0.1
Renal disease phenotype				99.4		0.6
Neurological phenotype				100.0		0.0
Cardiological/vision/hearing/Skeletal/maxillofacial deformity				100.0		0.0
Other extrarenal phenotypes			3.9	96.1		0.0
Genetic detection date		1.2		98.8		0.0
Genetic testing institutions				99.6		0.4
Sample type				99.5		0.5
GRCh37/38				94.9	4.6	0.5
Variants				99.8		0.2
SNP				99.9		0.1
chromosome				99.7		0.3
location				99.7		0.3
The reference sequence				96.4	3.5	0.1
Variation sequence				99.9		0.1
Genes symble				99.5		0.5
Variants type				99.6		0.4
Inheritance patterns				99.6		0.4
Variation category				99.5		0.5
transcript				99.7		0.3
exon				99.8		0.2
Nucleotide variation				99.6		0.4
Amino acid variation				99.6		0.4
Prediciton of pathogenicity				99.5		0.5
Segregation				92.2		7.8
Maternal/Paternal				91.6		8.4
